# m^6^A‐Modified circRAPGEF1 Interaction with IGF2BP3 Promotes Hepatocellular Carcinoma Progression via Reprogramming Aspartate Metabolism

**DOI:** 10.1002/advs.202503851

**Published:** 2025-08-13

**Authors:** Juanyi Shi, Sintim Mui, Yongcong Yan, Shaomin Liu, Kai Wen, Chuanchao He, Huoming Li, Hao Liao, Meng Tao, Jiahua Wen, Weidong Wang, Xiaoding Xu, Zhenyu Zhou, Zhiyu Xiao

**Affiliations:** ^1^ Guangdong Provincial Key Laboratory of Malignant Tumor Epigenetics and Gene Regulation Guangdong‐Hong Kong Joint Laboratory for RNA Medicine Yat‐Sen Memorial Hospital, Sun Yat‐Sen University Guangzhou 510120 China; ^2^ Department of Hepatobiliary Surgery Sun Yat‐Sen Memorial Hospital, Sun Yat‐Sen University Guangzhou 510120 China; ^3^ Department of Urology Sun Yat‐Sen Memorial Hospital, Sun Yat‐Sen University Guangzhou 510120 China; ^4^ Medical Research Center Sun Yat‐Sen Memorial Hospital, Sun Yat‐Sen University Guangzhou 510120 China; ^5^ Department of Interventional Radiology Sun Yat‐Sen Memorial Hospital, Sun Yat‐Sen University Guangzhou 510120 China

**Keywords:** aspartate metabolism, circular RNAs, hepatocellular carcinoma, liver cancer stem cells, m^6^A modification

## Abstract

Hepatocellular carcinoma (HCC) progression and therapy sensitivity are critically fueled by liver cancer stem cells (LCSCs), yet the regulatory mechanisms of circular RNAs (circRNAs) on LCSCs remain elusive. Here, through circRNA microarray analysis of LCSCs and non‐stem HCC cells, circRAPGEF1 is identified as a LCSC‐enriched circRNA upregulated in HCC tissues and predictive of poor patient survival. Functionally, circRAPGEF1 promoted the stemness properties, proliferation, and tumorigenicity of HCC cells. Mechanistically, the METTL3‐mediated *N^6^*‐methyladenosine (m^6^A) modification of circRAPGEF1 facilitated KH domain‐dependent binding of IGF2BP3 to its UGGAC motif, which conferring stability to circRAPGEF1 while competitively disrupting the IGF2BP3/*ASS1* mRNA interaction. This process led to the degradation of *ASS1* mRNA, triggering aspartate accumulation and activation of the S6K/CAD signaling pathway. Crucially, circRAPGEF1 overexpression reduced the sorafenib sensitivity, whereas targeting circRAPGEF1 using nanoparticles‐mediated systematic siRNAs delivery effectively sensitized HCC cells to sorafenib. Collectively, these findings unveil a METTL3/circRAPGEF1/IGF2BP3/ASS1 regulatory axis that drives aspartate metabolic reprogramming to fuel HCC stemness properties, positioning circRAPGEF1 as a dual prognostic biomarker and therapeutic target to enhance sorafenib efficacy in HCC.

## Graphical abstract

In hepatocellular carcinoma (HCC), the m^6^A‐modified circRAPGEF1 destabilizes *ASS1* mRNA via competitive binding to IGF2BP3, driving aspartate metabolic reprogramming in liver cancer stem cells (LCSCs). This cascade enhances stemness properties and reduces sorafenib sensitivity in LCSCs, thereby identifying circRAPGEF1 as a promising therapeutic target for HCC.

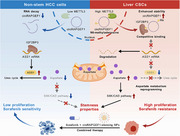

## 1 Introduction

Primary liver cancer ranks sixth in global cancer incidence and third in related mortality annually.^[^ ^1^ ^]^ More than 90% of primary liver cancer cases manifest as hepatocellular carcinoma (HCC). Despite curative surgery, early‐stage HCC exhibits a 70% recurrence rate within 5 years, while advanced HCC shows limited response to systemic therapies such as sorafenib, leading to an overall dismal prognosis.^[^ ^2^ ^]^ Liver cancer stem cells (LCSCs), a minor cellular subset whin heterogenous HCC cells, possess self‐renewal and multi‐lineage differentiation capacities.^[^ ^3^ ^]^ Among various stemness markers, CD133 (PROM1)‐positive cells are considered archetypal LCSCs, closely associated with HCC recurrence and treatment resistance.^[^ ^4^ ^]^ Hence, unraveling the molecular mechanisms underlying LCSC stemness regulation may provide novel therapeutic avenues for HCC.

Circular RNAs (circRNAs), characterized by covalently closed‐loop structures, are non‐coding RNAs with stable structures, high species conservation, and tissue‐specific expression.^[^ ^5^ ^]^ circRNAs can regulate tumor biology through various mechanisms, with *N^6^*‐methyladenosine (m^6^A) modification playing a crucial role.^[^ ^6^ ^]^ Several studies have implicated that m^6^A‐modified circRNAs promote the proliferation,^[^ ^7^ ^]^ invasion,^[^ ^8^ ^]^ and therapeutic resistance^[^ ^9^ ^]^ of HCC. However, the precise mechanism of m^6^A‐modified circRNAs in regulating LCSC stemness requires further elucidation.

Metabolic reprogramming exerts significant regulatory effects on tumorigenesis and progression, constituting a prominent hallmark of malignancies.^[^ ^10^ ^]^ Aspartate, a non‐essential amino acid, is primarily synthesized in mitochondria and transported to the cytoplasm, where it participates in the urea cycle or de novo pyrimidine synthesis.^[^ ^11^ ^]^ Increasing evidence indicates that many types of tumors exhibit dependence on aspartate. Restricting aspartate availability in tumor cells has been shown as a potential therapeutic approach.^[^ ^12^, ^13^, ^14^ ^]^ While elevated levels of aspartate have been observed in tumor tissues and peripheral blood of HCC patients,^[^ ^15^, ^16^ ^]^ yet the specific role of aspartate in the pathogenesis of HCC remains largely unclear.

In the present study, we identified that circRAPGEF1 is upregulated through a METTL3/IGF2BP3‐mediated m^6^A modification‐dependent manner in HCC and LCSCs. By decreasing the stability and expression of *ASS1* mRNA, circRAPGEF1 reprograms aspartate metabolism, thereby activating the S6K/CAD signaling pathway, enhancing HCC cell stemness properties and proliferation. Furthermore, we found that targeting circRAPGEF1 by siRNA‐loaded nanoparticles (NPs) sensitizes HCC cells to sorafenib therapy.

## 2 Results

### 2.1 Identification and Characteristics of circRAPGEF1 in HCC

**Figure 1:**
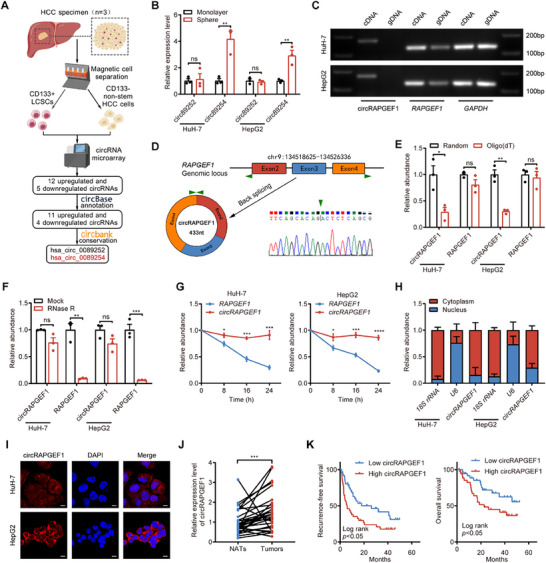
circRAPGEF1 is upregulated and associated with poor prognosis in HCC patients. A) Schematic diagram of circRNAs screening in CD133‐positive LCSCs. B) qRT‐PCR analysis of differentially expressed circRNAs expression in adherent monolayer cells and cell spheres of HCC cells. C) Agarose gel electrophoresis of PCR products with divergent and convergent primers in cDNA and gDNA amplification. D) Schematic diagram of the back‐splicing structure of circRAPGEF1 and Sanger sequencing results of PCR products. E) qRT‐PCR analysis of circRAPGEF1 and host gene *RAPGEF1* mRNA expression in reverse transcription with oligo (dT) or random primers. F) qRT‐PCR analysis of the circRAPGEF1 and *RAPGEF1* mRNA expression post‐RNase R treatment. G) qRT‐PCR analysis of circRAPGEF1 and *RAPGEF1* mRNA expression over time with Actinomycin D treatment (5 µg mL^−1^) in HCC cells. H) qRT‐PCR analysis of circRAPGEF1 expression in nucleus and cytoplasm of HCC cells. I) Representative FISH images of circRAPGEF1 in HCC cells. Scale bar: 10 µm. J) qRT‐PCR analysis of circRAPGEF1 expression in HCC tissues versus NATs (*n* = 30). K) Kaplan–Meier analysis of the recurrence‐free survival and overall survival of HCC patients with low versus high circRAPGEF1 expression (*n* = 74). The cutoff is the median. Data are presented as the means ± SD and analyzed by Student's *t*‐test. * p < 0.05; ** p < 0.01; *** p < 0.001; **** p < 0.0001; ns: not significant.

To screen for aberrantly expressed circRNAs in LCSCs, we analyzed paired CD133‐positive LCSCs and CD133‐negative non‐stem HCC cells isolated from three clinical HCC specimens using circRNA microarray. Among the differentially expressed circRNAs (|Fold change| > 1.5, *p* < 0.05), has_circ_0089252 and has_circ_0089254 were annotated by the circBase^[^ ^17^ ^]^ database and confirmed for species conservation by the circBank^[^ ^18^ ^]^ database (**Figure** **1**A). Notably, only hsa_circ_0089254 exhibited significant upregulation in HCC cell spheres compared with adherent cells (Figure 1B), suggesting its association with the stemness properties of HCC. Therefore, we selected has_circ_0087394 for further validation.

The structure analysis revealed that the hsa_circ_0089254 (termed circRAPGEF1) is generated through the back‐splicing of exons 2, 3, and 4 of the host gene *RAPGEF1*, with a length of 433 nt. The covalently closed circular structure of circRAPGEF1 was verified by PCR with divergent and convergent primers. Nucleic acid electrophoresis of the PCR products demonstrated that the circRAPGEF1 could be amplified by divergent primers in cDNA but not in gDNA of HCC cells (Figure 1C). Additionally, Sanger sequencing of the PCR products confirmed the back‐splicing junction (BSJ) of circRAPGEF1 (Figure 1D). Reverse transcription assays demonstrated that circRAPGEF1 could not be reverse transcribed by oligo(dT) primers (Figure 1E), indicating that circRAPGEF1 lacks a poly A‐tail. RNase R digestion and RNA degradation assays revealed a significant reduction in linear mRNA compared with circRAPGEF1 (Figure 1F,G), suggesting that circRAPGEF1 exhibits enhanced stability. RNA subcellular isolation assays demonstrated that circRAPGEF1 was predominantly localized in the cytoplasm of HCC cells (Figure 1H), which were corroborated by the FISH assays of HCC cells and clinical specimens (Figure 1I; Figure S1A, Supporting Information).

We then analyzed the expression profile of circRAPGEF1 from the MiOncoCirc database and found that the presence of circRAPGEF1 was particularly high expressed in HCC across a variety of tumors (Figure S1B, Supporting Information). To elucidate clinical relevance of circRAPGEF1 in HCC, we the examined expression levels of circRAPGEF1 in HCC specimens and normal adjacent tissues (NATs) using qRT‐PCR. The results demonstrated that circRAPGEF1 was significantly upregulated in HCC specimens compared with NATs (*p* = 0.0005; Figure 1J; Table S1, Supporting Information). Notably, HCC patients with high circRAPGEF1 expression exhibited reduced recurrence‐free survival (*p* = 0.0171) and overall survival (*p* = 0.0239) compared with those with low circRAPGEF1 expression (Figure 1K). Collectively, these results demonstrate that stemness‐associated circRAPGEF1 is closely related to the poor prognosis of patients with HCC.

### 2.2 circRAPGEF1 Promotes Stemness Properties and Proliferation of HCC Cells

**Figure 2:**
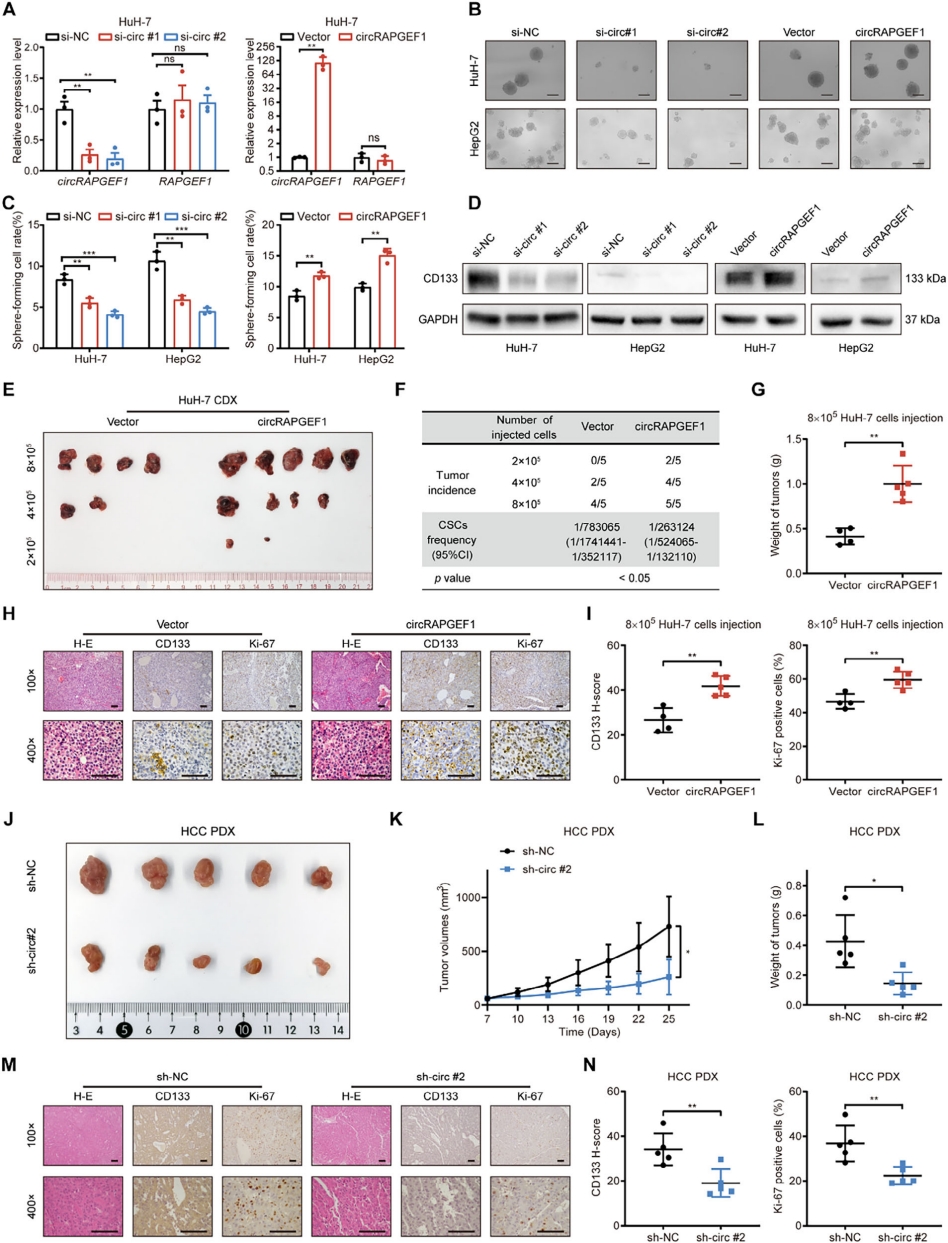
circRAPGEF1 enhances stemness properties and proliferation in HCC. A) qRT‐PCR analysis of circRAPGEF1 and *RAPGEF1* expression in HuH‐7 cells transfected with control or circRAPGEF1‐targeting siRNAs and Vector or circRAPGEF1 overexpression lentivirus. B,C) Representative images and quantification of tumor sphere formation assays in HCC cells with the indicated treatments. Scale bar: 100 µm. D) Western blot of CD133 expression in HCC cells with the indicated treatments. E) Image of HCC tumors formed after subcutaneous implantation of different numbers of stably overexpressed circRAPGEF1 and control HuH‐7 cells in BALB/c nude mice (*n* = 5). F) Extreme limited dilution analysis for LCSC proportions. G) Weights of subcutaneous HCC tumors in the 8 × 10^5^ HuH‐7 cell implantation group. H,I) Representative images and quantification of IHC staining for H&E, CD133, and Ki‐67 in tumor specimens. Scale bar: 100 µm. J–L) Image, volumes, and weights of PDX tumors in mice with the indicated treatments (*n* = 5). M,N) Representative images and quantifications of IHC staining of H&E, Ki‐67, and CD133 in PDX specimens. Scale bar: 100 µm. Data are presented as mean ± SD and analyzed by Student's *t*‐test or one‐way ANOVA with Tukey's multiple comparison test. * p < 0.05; ** p < 0.01; *** p < 0.001; ns: not significant.

To elucidate the biological function of circRAPGEF1, we specifically knocked down circRAPGEF1 using siRNAs targeting the BSJ of circRAPGEF1, and overexpressed circRAPGEF1 via lentiviral transfection in HuH‐7 and HepG2 cells. Notably, these manipulations did not affect the expression of linear *RAPGEF1* mRNA (**Figure** **2**A; Figure S2A, Supporting Information). Sphere formation assays demonstrated that circRAPGEF1 silencing significantly reduced the number of spheres, while circRAPGEF1 overexpression markedly enhanced the sphere formation capacity (Figure 2B,C). Western blot analysis revealed that circRAPGEF1 modulated the expression levels of stemness marker CD133 in HCC cells (Figure 2D). To further demonstrate the oncogenic potential of circRAPGEF1 in HCC, we conducted an in vivo limiting dilution assay. The results indicated that circRAPGEF1 overexpression increased the proportion of LCSCs and promoted tumor growth compared with the control cells (Figure 2E–G; Figure S2H,I, Supporting Information). immunohistochemical (IHC) analysis demonstrated that circRAPGEF1 overexpression resulted in higher CD133 expression level and proportion of Ki‐67‐positive cells in HCC (Figure 2H,I).

Additionally, we found that circRAPGEF1 silencing significantly inhibited HCC cell proliferation, whereas circRAPGEF1 overexpression markedly enhanced proliferative capacity, as demonstrated by CCK‐8 and colony formation assays (Figure S2B–E, Supporting Information). EdU assays indicated that circRAPGEF1 silencing reduced the proportion of EdU‐positive cells, while circRAPGEF1 overexpression increased it in HCC cells (Figure S2F,G, Supporting Information). To further substantiate the pivotal role of circRAPGEF1 in HCC progression, we established a patient‐derived xenograft (PDX) model of HCC. Intratumoral injection of lentivirus targeting circRAPGEF1 significantly inhibited PDX tumor growth (Figure 2J–L). IHC analysis demonstrated that the circRAPGEF1 silencing reduced CD133 expression and the proportion of Ki‐67‐positive cells (Figure 2M,N). Collectively, these results demonstrate that circRAPGEF1 promotes stemness properties and cell proliferation of HCC cells in vitro and in vivo.

### 2.3 Investigation of the Interaction Between circRAPGEF1 and IGF2BP3

**Figure 3:**
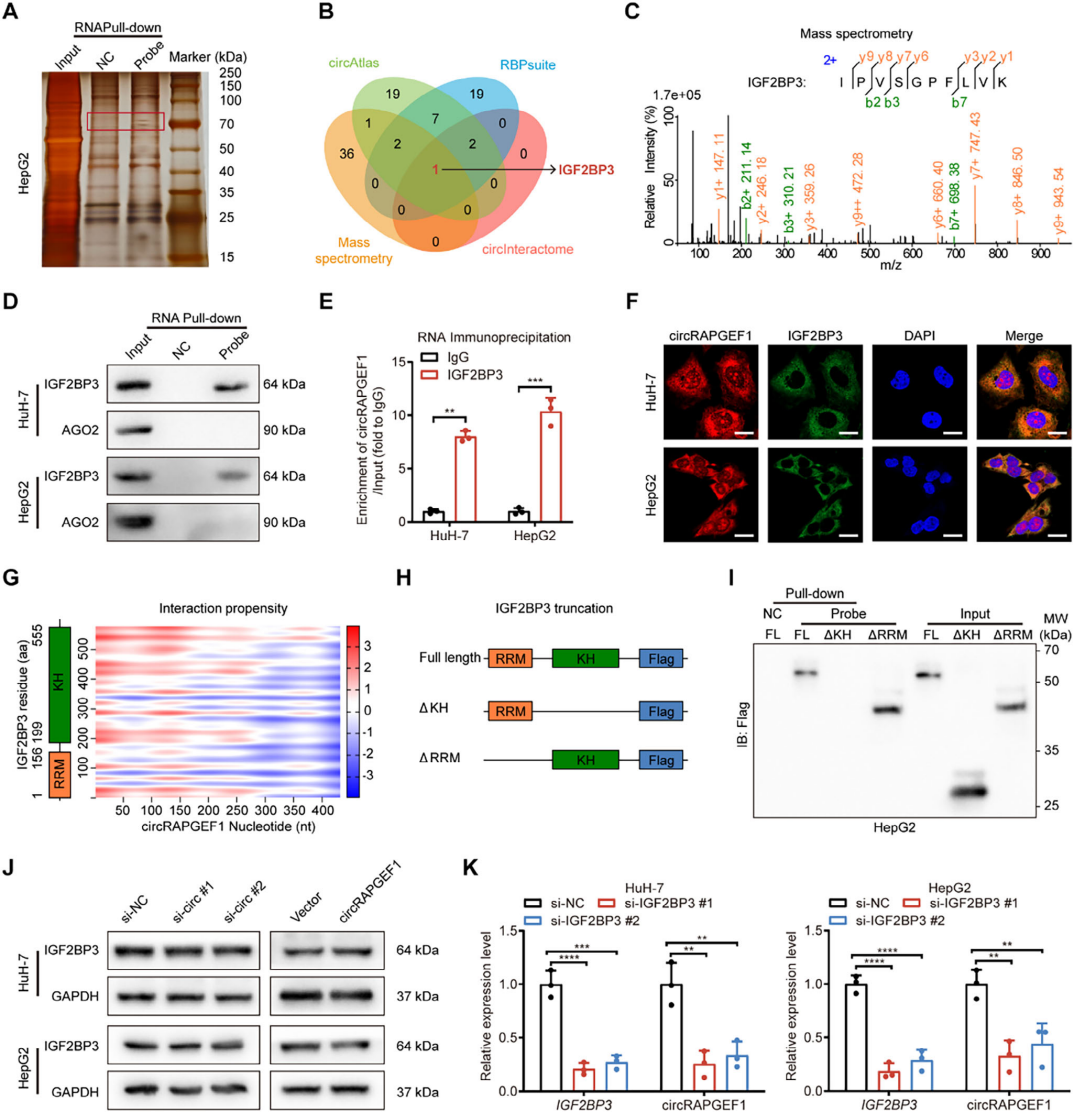
circRAPGEF1 interacts with IGF2BP3 in HCC cells. A) Silver staining image of RNA pull‐down assay with circRAPGEF1 and negative control (NC) probes. B) Venn diagram of potential RBPs binding circRAPGEF1 in mass spectrometry analysis and databases. C) Mass spectrometry analysis of IGF2BP3 peptides pulled down by circRAPGEF1 probe. D) Western blot analysis of the interaction between IGF2BP3 and circRAPGEF1 in HCC cells post RNA pull‐down assays. E) qRT‐PCR analysis of circRAPGEF1 enrichment in IGF2BP3 RIP assays. F) Representative images of circRAPGEF1 (Red) and IGF2BP3 (Green) co‐localization in HCC cells. Scale bar: 20 µm. G) Diagram predicting IGF2BP3 and circRAPGEF1 interaction via catRAPID. H) Schematic diagram of IGF2BP3 structural domain truncated constructs. I) Western blot analysis of Flag‐labeled IGF2BP3 truncations enrichment post RNA pull‐down assays in HepG2 cells. J) Western Blot analysis of IGF2BP3 expression in HCC cells with circRAPGEF1 silencing or overexpression. K) qRT‐PCR analysis of *IGF2BP3* and circRAPGEF1 expression in HCC cells transfected with *IGF2BP3*‐targeting siRNAs. Data are presented as mean ± SD and analyzed by Student's *t*‐test or one‐way ANOVA with Tukey's multiple comparison test. ** p < 0.01; *** p < 0.001; ns: not significant.

It has been proposed that circRNAs exert biological functions by binding RNA‐binding proteins (RBPs). To identify RBPs interacting with circRAPGEF1, we performed RNA pull‐down assays followed by mass spectrometry in HuH‐7 cell (**Figure** **3**A). The results corroborated in silico predictions by the CircAtlas,^[^ ^19^ ^]^ RBPsuite,^[^ ^20^ ^]^ and circInteractome^[^ ^21^ ^]^ databases, suggesting IGF2BP3 as a potential interactor with circRAPGEF1 (Figure 3B,C; Table S2, Supporting Information). RNA pulldown assays confirmed the interaction between circRAPGEF1 and IGF2BP3 in HCC cells (Figure 3D), while RNA immunoprecipitation (RIP) assays showed that circRAPGEF1 was significantly enriched in IGF2BP3 antibody compared with IgG (Figure 3E). Moreover, FISH combined with IF confirmed that the interaction between circRAPGEF1 and IGF2BP3 occurred in the cytoplasm of HCC cells (Figure 3F; Figure S3, Supporting Information). Previous studies have indicated that IGF2BP3 contains two canonical RNA‐binding domains: the RNA recognition motif (RRM) and the K homology (KH) domain.^[^ ^22^ ^]^ Using the catRAPID^[^ ^23^ ^]^ tool, we predicted a stronger interaction between circRAPGEF1 and the KH domain of IGF2BP3 (Figure 3G). To delineate the precise binding sites, we constructed Flag‐tagged full‐length IGF2BP3 (FL), KH domain‐depleted (ΔKH), and RRM domain‐depleted (ΔRRM) plasmids (Figure 3H). RNA pull‐down assays confirmed specific binding of circRAPGEF1 to FL and ΔRRM but not to ΔKH (Figure 3I), indicating an interaction with the KH domain. We then investigated the regulatory dynamics arising from the interaction between circRAPGEF1 and IGF2BP3. We found that silencing or overexpression of circRAPGEF1 did not alter IGF2BP3 expression levels (Figure 3J), whereas IGF2BP3 knockdown significantly reduced circRAPGEF1 expression (Figure 3K). Together, we identified IGF2BP3 as a binding partner of circRAPGEF1, and found that this binding can regulate the expression of circRAPGEF1.

### 2.4 METTL3/IGF2BP3 Axis Medicates m^6^A Modification of circRAPGEF1

**Figure 4:**
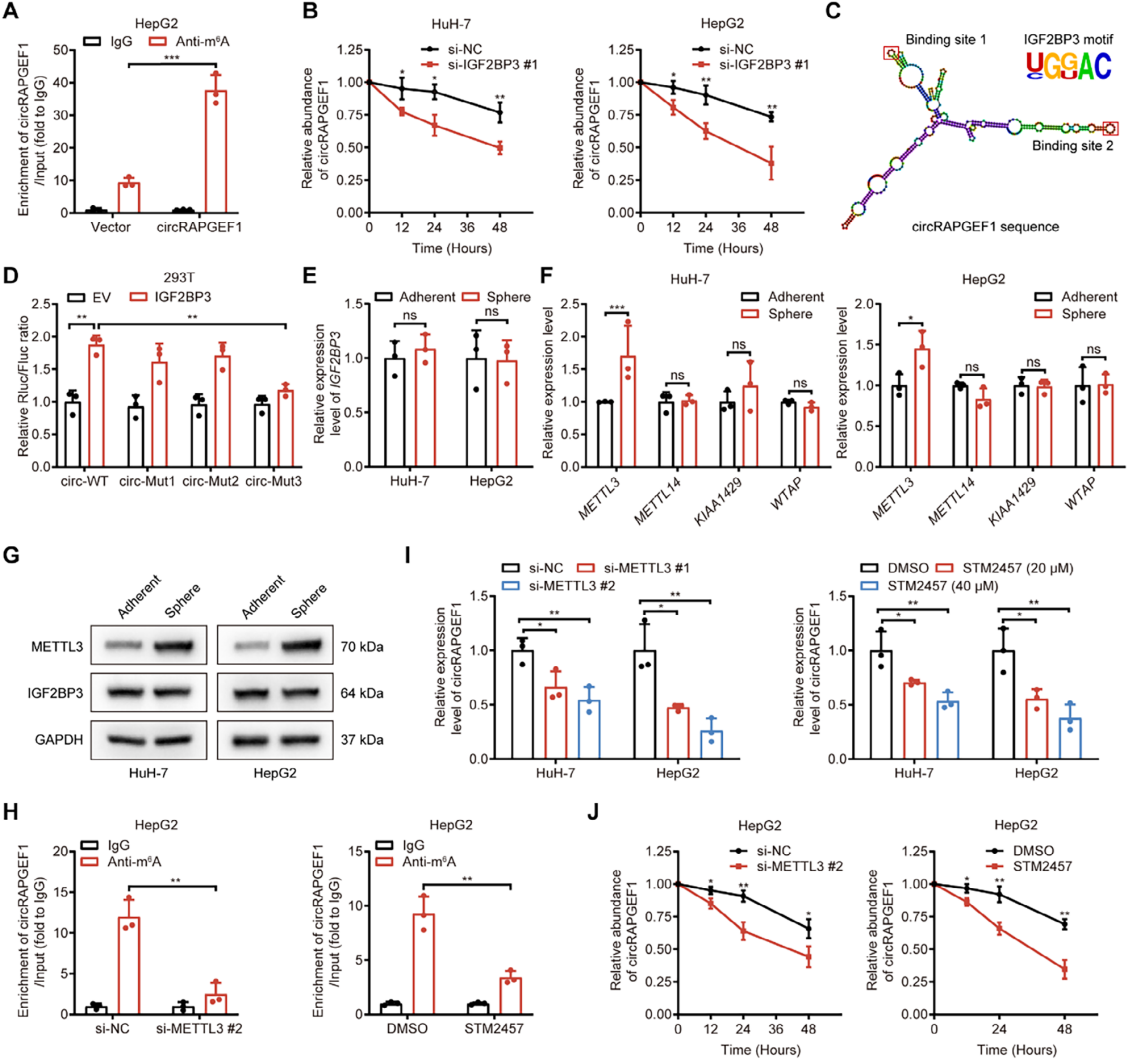
METTL3/IGF2BP3 enhances the stability of m^6^A‐modified circRAPGEF1. A) qRT‐PCR analysis of circRAPGEF1 enrichment in m^6^A RIP assays in HCC cells. B) qRT‐PCR analysis of the circRAPGEF1 expression over time in HCC cells transfected with *IGF2BP3*‐targeting siRNAs. C) Schematic diagram of circRAPGEF1 sequence labeled with IGF2BP3‐recognizing m^6^A motif. D) Relative luciferase activity of HEK‐293T cells with the indicated treatments. E–G) qRT‐PCR and Western Blot analysis of IGF2BP3 and METTL3 expression in adherent cells and sphere cells of HCC cells. H) qRT‐PCR analysis of circRAPGEF1 enrichment in IGF2BP3 RIP assays in HepG2 cells with METTL3 silencing or STM2457 treatment (40 µm). I,J) qRT‐PCR analysis of circRAPGEF1 expression in HCC cells with the indicated treatments. Data are presented as mean ± SD and analyzed by Student's *t*‐test or one‐way ANOVA with Tukey's multiple comparison test. * p < 0.05; ** p < 0.01; *** p < 0.001; ns: not significant.

IGF2BP3, a member of the insulin‐like growth factor‐2 mRNA‐binding protein family, is known to stabilize RNAs by recognizing m^6^A modifications.^[^ ^24^ ^]^ Bioinformatics analysis (Figure S4A, Supporting Information) and m^6^A RIP (**Figure** **4**A) identified m^6^A modifications in circRAPGEF1. RNA decay assays indicated that silencing IGF2BP3 significantly decreased circRAPGEF1 stability (Figure 4B), suggesting that IGF2BP3 stabilizes circRAPGEF1 via recognition of its m^6^A modifications. Furthermore, we cloned sequences containing wild‐type IGF2BP3‐recognizing m^6^A motifs (WT), single mutants (mut1 and mut2), and double mutants (mut3) into the psiCHECK2 vector (Figure 4C; Figure S4B, Supporting Information). Dual fluorescence reporter assays demonstrated that IGF2BP3 overexpression significantly increased fluorescence in cells transfected with WT, mut1, and mut2 vectors, but not with mut3 vector (Figure 4D). These results suggested IGF2BP3 regulated circRAPGEF1 stability through binding to both m^6^A motifs of circRAPGEF1.

Unexpectedly, IGF2BP3 expression was unaltered in HCC cell spheres compared with adherent cells, whereas METTL3, the key methyltransferase of m^6^A writer complex, was upregulated in both HuH‐7 and HepG2 cell spheres (Figure 4E–G). TCGA data indicated that elevated METTL3 expression in HCC tissues compared with normal liver tissues (Figure S5A, Supporting Information), and high METTL3 expression was associated with poor prognosis (Figure S5B, Supporting Information) and positively correlated with expression of stemness markers including CD133 (Figure S5C–H, Supporting Information). Interestingly, silencing METTL3 or treating HCC cells with the METTL3 inhibitor STM2457 resulted in a significant reduction in the binding of IGF2BP3 to circRAPGEF1(Figure 4H), accompanied by a decrease in circRAPGEF1 expression and stability (Figure 4I,J). Taken together, these results indicate that circRAPGEF1 in HCC was stabilized in a m^6^A‐dependent manner by METTL3/IGF2BP3 axis.

### 2.5 circRAPGEF1 Destabilizes *ASS1* mRNA in an IGF2BP3‐Dependent Manner

**Figure 5:**
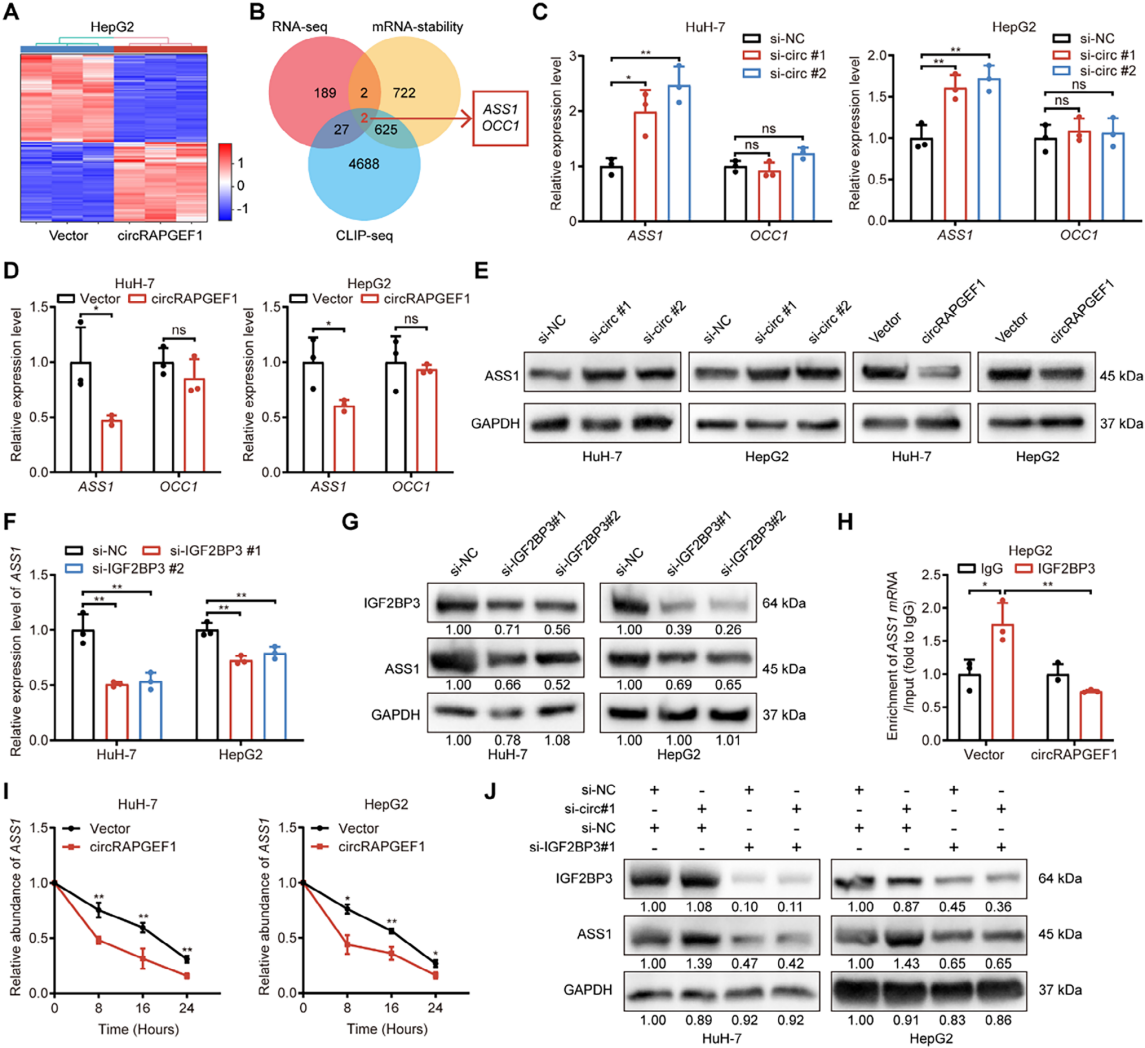
circRAPGEF1 decreases the stability of *ASS1* mRNA in HCC. A) Heatmap of DEGs in HepG2 cells with control or circRAPGEF1 overexpression. B) A Venn diagram showing the co‐regulated genes by circRAPGEF1 and IGF2BP3 in GSE90684 and ENCORI database. C–F) qRT‐PCR analysis of *ASS1* and *OCC1* expression in HCC cells with circRAPGEF1 silencing (C,D) and overexpression (E,F). G) Western blot analysis of ASS1 expression in HCC cells with the indicated treatments. H) qRT‐PCR analysis of *ASS1* mRNA enrichment in IGF2BP3 RIP assay in HepG2 cells. I) qRT‐PCR analysis of *ASS1* mRNA expression over time in HCC cells with the indicated treatments. J) Western blot analysis of ASS1 expression in HCC cells with the indicated treatments. Data are presented as mean ± SD and analyzed by Student's *t*‐test or one‐way ANOVA with Tukey's multiple comparison test. * p < 0.05; ** p < 0.01; ns: not significant.

To elucidate the precise mechanism by which circRAPGEF1 promotes HCC progression, we performed RNA‐seq to identify differentially expressed genes (DEGs) regulated by circRAPGEF1 (**Figure** **5**A). Comprehensive analysis of these DEGs, integrating with IGF2BP3‐related mRNA stability data (GSE90684) and IGF2BP3 binding mRNA from CLIP assay (ENCORI database), highlighted *ASS1* and *OCC1* as potential targets (Figure 5B). Subsequent qRT‐PCR validation revealed that only *ASS1* expression was regulated by circRAPGEF1, with no effect observed on *OCC1* (Figure 5C,D). Western blot further confirmed that circRAPGEF1 knockdown increased ASS1 expression, while and circRAPGEF1 overexpression decreased ASS1 expression (Figure 5E). Nonetheless, analogous to circRAPGEF1, the expression of ASS1 was found to be positively regulated by IGF2BP3 (Figure 5F,G). These results prompted us to speculate that circRAPGEF1 might destabilize *ASS1* mRNA through its interaction with IGF2BP3.

RIP assays demonstrated that circRAPGEF1 overexpression decreased IGF2BP3 binding affinity to *ASS1* mRNA in HepG2 cells (Figure 5H). RNA decay assays further revealed that circRAPGEF1 overexpression destabilized *ASS1* mRNA in HCC cells (Figure 5I). Additionally, we investigated the regulatory effect of circRAPGEF1 on ASS1 in the context of IGF2BP3 presence or absence. Notably, IGF2BP3 knockdown significantly mitigated the suppressive impact of circRAPGEF1 on ASS1 expression (Figure 5J). Collectively, these results indicate that circRAPGEF1 competes with *ASS1* mRNA for binding to IGF2BP3, resulting in the destabilization of *ASS1* mRNA and subsequent downregulation of ASS1 expression in HCC cells.

### 2.6 circRAPGEF1 Promotes HCC Progression Through Downregulating ASS1 Expression

**Figure 6:**
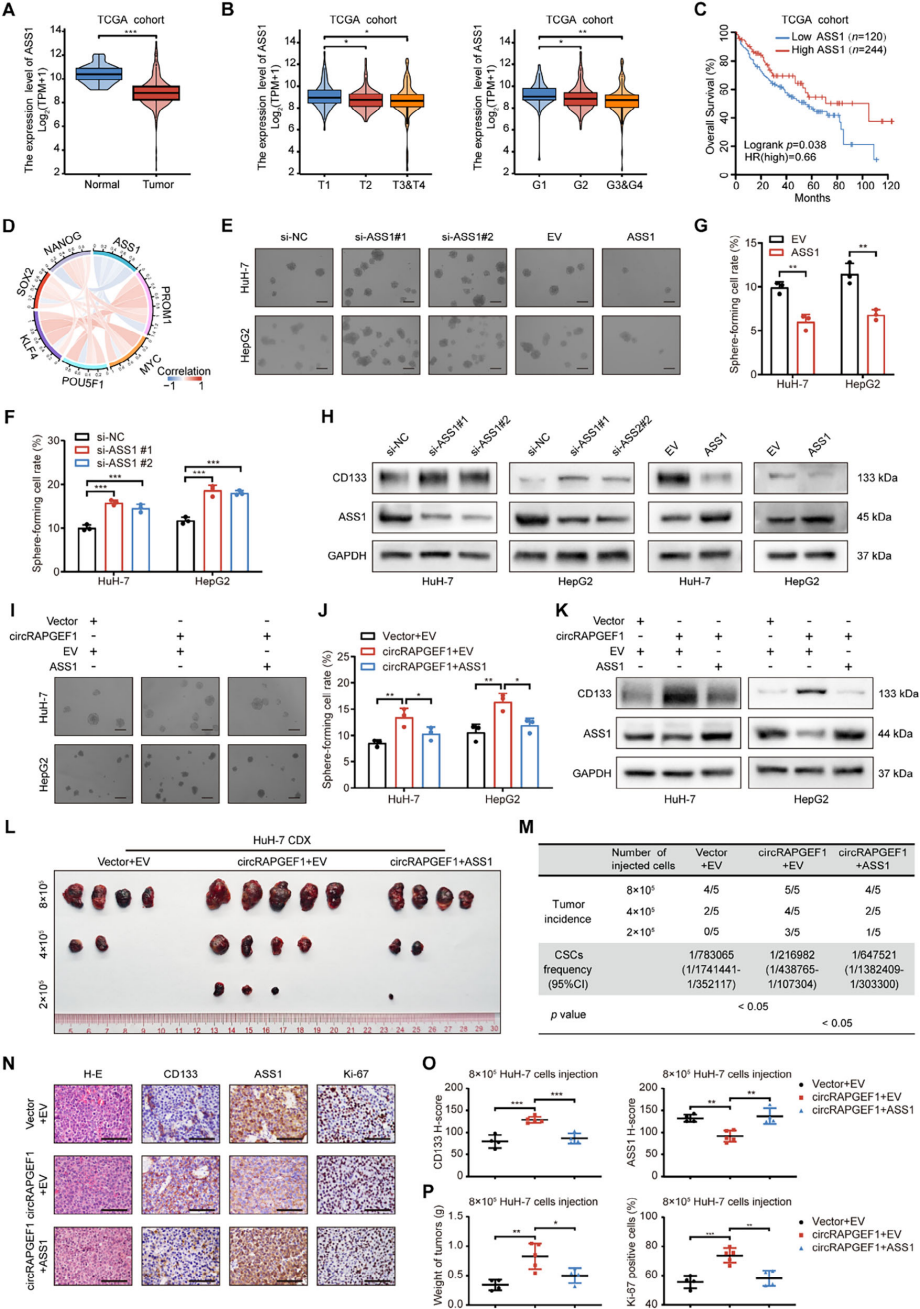
circRAPGEF1 downregulates ASS1 to enhance stemness properties in HCC. A–C) Boxplots illustrating the ASS1 expression (A,B) and K‐M curve analysis of OS (C) in TCGA‐LIHC cohort. D) Chord diagram illustrating the correlation between ASS1 and stemness markers. E–G) Representative images and quantification of tumor sphere formation assays in HCC cells with ASS1 silencing or overexpression. Scale bar: 100 µm. H) Western blot analysis of CD133 and ASS1 expression of HCC cells with the indicated treatments. I,J) Representative images and quantification of tumor sphere formation assays in HCC cells co‐transfected with vector/circRAPGEF1 overexpression lentivirus and empty vector (EV)/ASS1 overexpression plasmid. Scale bar: 100 µm. K) Western blot analysis of CD133 and ASS1 expression in rescue assay. L) Image of HCC tumors formed after subcutaneous implantation of different numbers of HCC cells with the indicated treatments in BALB/c nude mice (*n* = 5). M) Extreme limited dilution analysis for LCSC proportions. N–P) Representative images and quantifications of IHC staining of H&E, CD133, ASS1, and Ki‐67, and weights of subcutaneous HCC tumors in the 8 × 10^5^ HuH‐7 cell implantation group. Data are presented as mean ± SD and analyzed by Student's *t*‐test or one‐way ANOVA with Tukey's multiple comparison test. * p < 0.05; ** p < 0.01; *** p < 0.001; ns: not significant.

ASS1 dysregulation has been implicated in various tumors and plays a crucial role in HCC progression.^[^ ^25^ ^]^ Our comprehensive multi‐omics analysis with our center cohort and public databases demonstrated a significant downregulation of ASS1 expression in HCC (**Figure** **6**A,B; Figure S6A–G, Supporting Information), and ASS1 expression was associated with favorable prognosis (Figure 6C) and negatively correlated with expression of stemness markers (Figure 6D; Figure S6H–M, Supporting Information). Sphere formation assays demonstrated that ASS1 silencing markedly enhanced sphere formation capacity, while ASS1 overexpression had the opposite effect (Figure 6E–G). Western blot analysis demonstrated that ASS1 downregulation reduced CD133 expression in HCC cells (Figure 6H). Additionally, functional assays revealed that ASS1 silencing promoted, while ASS1 overexpression inhibited HCC cell proliferation, as evidenced by CCK‐8, colony formation, and EdU assays (Figure S7A–F, Supporting Information).

To validate the role of ASS1 in circRAPGEF1‐mediated HCC progression, rescue assays were performed. Overexpression of ASS1 in HCC cells abrogated the sphere formation enhancement induced by circRAPGEF1 (Figure 6I–J). Western blot analysis revealed that ASS1 overexpression impeded the circRAPGEF1‐induced CD133 expression (Figure 6K). Furthermore, an in vivo limiting dilution assay was conducted in order to provide additional evidence for the function of ASS1 on the circRAPGEF1 in HCC. The results indicated that ASS1 overexpression reduced the proportion of LCSCs in comparison with cells overexpressing circRAPGEF1 (Figure 6L,M). IHC analysis demonstrated that ASS1 overexpression resulted in a decrease in CD133 expression level of HCC cells induced by circRAPGEF1 overexpressing (Figure 6N,O). Also, ASS1 overexpression reversed the pro‐proliferative effect of circRAPGEF1 on HCC cells (Figure S8A–E, Supporting Information; Figure 6P). These findings collectively underscore the role of ASS1 in attenuating the circRAPGEF1‐induced stemness properties and proliferation of HCC cells.

### 2.7 circRAPGEF1‐Mediated Aspartate Accumulation Augments Malignancies in HCC Cells via S6K/CAD Pathway

**Figure 7:**
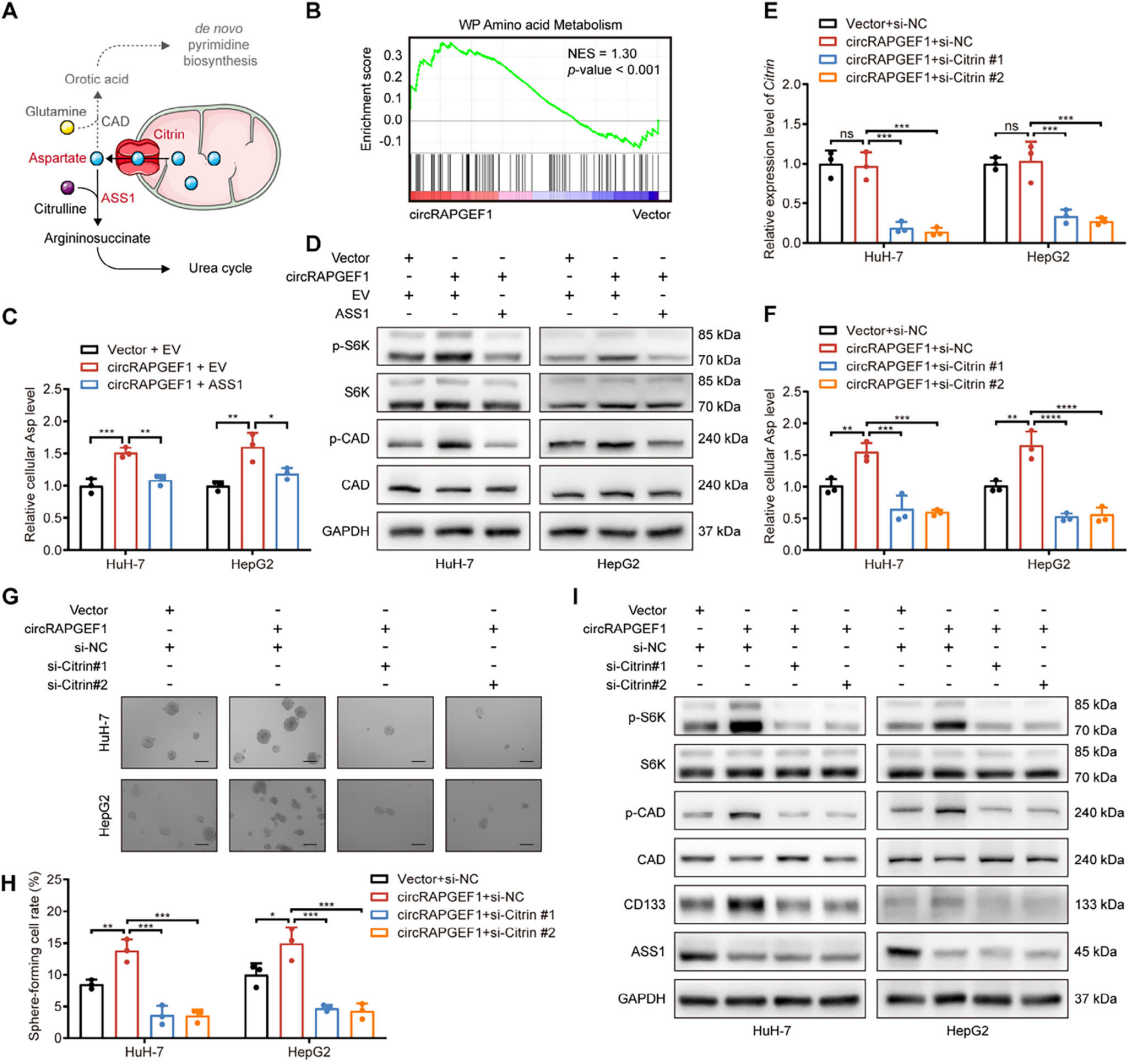
circRAPGEF1 upregulates aspartate levels to enhance stemness properties in HCC cells. A) Schematic diagram of aspartate metabolism in cells. B) GSEA of amino acid metabolism‐related genes in HepG2 cells with vector and circRAPGEF1 overexpression. C) Intracellular aspartate levels of HCC cells co‐transfected with vector/circRAPGEF1 overexpression lentivirus and EV/ASS1 overexpression plasmid. D) Western blot analysis of p‐S6K, S6K, p‐CAD, and CAD expression of HCC cells with the indicated treatments. E) qRT‐PCR analysis of *Citrin* expression in HCC cells co‐transfected with vector/circRAPGEF1 overexpression lentivirus and negative control/*Citrin*‐targeting siRNAs. F) Intracellular aspartate levels of HCC cells with the indicated treatments. G,H) Representative images and quantification of tumor sphere formation assays in HCC cells with the indicated treatments. Scale bar: 100 µm. I) Western blot analysis of p‐S6K, S6K, p‐CAD, CAD, CD133, and ASS1 expression in HCC cells with the indicated treatments. Data are presented as mean ± SD and analyzed by one‐way ANOVA with Tukey's multiple comparison test. * p < 0.05; ** p < 0.01; *** p < 0.001; **** p < 0.0001; ns: not significant.

ASS1 is a key enzyme in aspartate metabolism (**Figure** **7**A) and serves a regulatory function in the intracellular aspartate levels.^[^ ^26^, ^27^ ^]^ Previous study has shown that aspartate promotes tumor growth through activating the S6K/CAD pathway.^[^ ^12^ ^]^ Besides, our published data have linked CAD with stemness properties and HCC progression.^[^ ^28^ ^]^ As expected, GSEA demonstrated that circRAPGEF1 overexpression in HepG2 cells upregulated amino acid metabolism pathway (Figure 7B). Aspartate assays indicated that circRAPGEF1 overexpression increased intracellular aspartate levels, which were neutralized by ASS1 overexpression (Figure 7C). Furthermore, circRAPGEF1 overexpression elevated phosphorylated S6K and CAD levels, which were mitigated by ASS1 overexpression (Figure 7D).

We then blocked aspartate metabolism in HCC cells by silencing the mitochondrial aspartate transporter, Citrin (Figure 7E). Citrin silencing effectively reduced the aspartate levels elevated by circRAPGEF1 overexpression (Figure 7F), and abrogated circRAPGEF1‐induced enhancement of stemness properties (Figure 7G,H) and proliferative capacity (Figure S9A–E, Supporting Information). Notably, blocking aspartate metabolism also negated the circRAPGEF1‐mediated activation of the S6K/CAD signaling pathway (Figure 7I). In addition, the relationship between circRAPGEF1 and S6K/CAD activation in HCC cells was further elucidated through the utilization of a S6K inhibitor, PF‐4708671 (Figure S9F, Supporting Information). Collectively, our findings suggest that circRAPGEF1 reprograms aspartate metabolism, subsequently activating the S6K/CAD signaling pathway and exacerbating the malignant phenotype of HCC cells.

### 2.8 NPs‐Mediated circRAPGEF1 Silencing Enhances the Sorafenib Sensitivity of HCC

**Figure 8:**
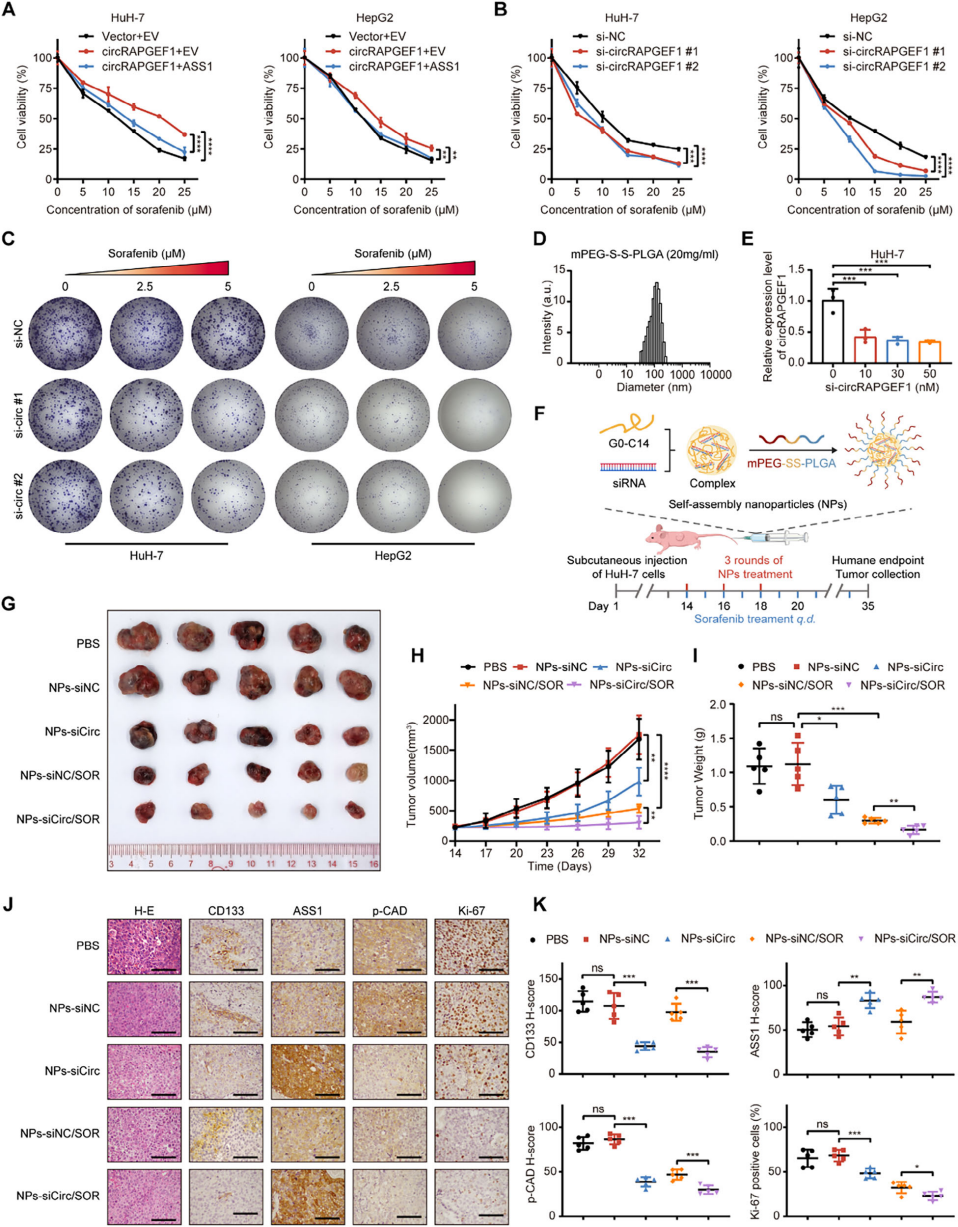
NPs‐mediated circRAPGEF1 silencing enhances sorafenib efficacy in HCC. A) CCK‐8 assay determined the inhibitory efficacy of sorafenib in HCC cells co‐transfected with vector/circRAPGEF1 overexpression lentivirus and EV/ASS1 overexpression plasmid. B,C) CCK‐8 assay and colony formation assay determined the inhibitory efficacy of sorafenib in HCC cells transfected with control or circRAPGEF1‐targeting siRNAs. D) Size distribution of NPs‐siCirc. E) qRT‐PCR analysis of circRAPGEF1 expression in HuH‐7 cells with control or NPs‐siCirc treatment. F) Schematic diagram for the in vivo validation of the combined treatment of NPs‐siCirc and sorafenib. G–I) Image, volumes, and weights of subcutaneous tumors of mice treated with PBS, NPs‐siNC, NPs‐siCirc, NPs‐siNC/Sorafenib (SOR), and NPs‐siCirc/SOR (*n* = 5). J, K) Representative images and quantification of IHC staining for ASS1, p‐CAD, CD133, and Ki‐67 in tumor specimens. Scale bar: 100 µm. Data are presented as mean ± SD and analyzed by one‐way ANOVA with Tukey's multiple comparison test. * p < 0.05; ** p < 0.01; *** p < 0.001; **** p < 0.0001; ns: not significant.

Previous studies have proposed that LCSCs exhibit heightened resistance to sorafenib^[^ ^29^ ^]^ and that the response of HCC patients to sorafenib treatment associates with CD133 expression within the tumor.^[^ ^30^ ^]^ We hypothesized that circRAPGEF1 might influence sorafenib sensitivity in HCC. Our results showed that circRAPGEF1‐overexpressing HCC cells displayed reduced sensitivity to sorafenib compared with control cells, whereas ASS1 overexpression partially restored sorafenib sensitivity (**Figure** **8**A). Moreover, circRAPGEF1 silencing significantly enhanced sensitivity to sorafenib in HCC cells (Figure 8B,C).

NPs have proven to be a safe and effective vehicle for delivering siRNAs.^[^ ^31^ ^]^ In previous work,^[^ ^32^ ^]^ we developed glutathione‐responsive nanoparticles, Meo‐PEG‐S‐S‐PLGA, capable of encapsulating siRNAs for effective tumor treatment.^[^ ^33^, ^34^ ^]^ In this study, we formulated circRAPGEF1‐siRNA‐loaded‐NPs (termed NPs‐siCirc) to evaluate the combined effects of circRAPGEF1 silencing and sorafenib in HCC treatment. Characteristics analysis indicated that the mean diameter of NPs was ≈100 nm (Figure 8D). In vitro experiments confirmed that the NPs‐siCirc effectively silenced circRAPGEF1 at a siRNA concentration of 30 nM (Figure 8E). Next, in vivo experiments were conducted to assess the therapeutic efficacy of NPs‐siCirc in combination with sorafenib using a subcutaneous tumor model in nude mice. Tumor‐bearing mice received intravenous injections of NPs‐siNC or NP‐siCirc every two days (*n* = 3, 1 nmol siRNA per mouse) and were administered sorafenib orally (20 mg kg^−1^) once daily (Figure 8F). As illustrated in Figure 8G–I, both NPs‐siCirc and sorafenib alone exhibited moderately inhibited tumor growth compared with the PBS control group and the NPs‐siNC group. Notably, the combination of sorafenib and NPs‐siCirc demonstrated the most pronounced therapeutic effect (Figure 8G–I). qRT‐PCR and IHC analysis revealed that NPs‐siCirc significantly reduced the expression of circRAPGEF1, ASS1, p‐CAD, and CD133 in tumors (Figure S10A, Supporting Information; Figure 8J,K). The combination therapy group exhibited the lowest percentage of Ki‐67 positivity compared with NPs‐siCirc or sorafenib alone (Figure 8K). No discernible histologic lesion was observed in the major organs (heart, liver, spleen, lungs, kidneys) of mice treated with NPs (Figure S10B, Supporting Information). Furthermore, blood tests indicated that transaminases and creatinine levels remained within the normal range for mice across all treatment groups (Figure S10C, Supporting Information). Taken together, these results propose that the combination of circRAPGEF1‐targeting NPs and sorafenib represents a promising strategy for HCC treatment with good biocompatibility.

## 3 Discussion

LCSCs have been demonstrated to possess heightened malignancy in HCC.^[^ ^35^ ^]^ Previous studies have highlighted the involvement of circRNAs in regulating HCC progression.^[^ ^36^ ^]^ However, the crucial roles of circRNAs in maintaining LCSC stemness remains largely unexplored. In the present study, we isolated paired CD133+ CSCs and CD133‐ non‐stem HCC cells from clinical HCC specimens. Following a series of screening processes, we confirmed that circRAPGEF1 is highly expressed in HCC and LCSCs, and is associated with poor prognosis in HCC patients. circRAPGEF1 promotes stemness properties and proliferation in HCC cells both in vitro and in vivo. Mechanistically, circRAPGEF1 is upregulated through a METTL3/IGF2BP3‐mediated m^6^A modification mechanism. circRAPGEF1 disrupts the stability of *ASS1* mRNA by competitively binding to IGF2BP3, leading to aspartate accumulation and activation of the S6K/CAD signaling pathway in HCC cells. More importantly, we found that targeting circRAPGEF1 with NPs can enhance the sensitivity of HCC cells to sorafenib treatment.

Tumor cell heterogeneity is regulated by epigenetic mechanisms, including m^6^A modifications.^[^ ^37^ ^]^ Dysregulation of m^6^A writer METTL3,^[^ ^38^ ^]^ readers YTHDF1/2^[^ ^39^, ^40^ ^]^ and IGF2BP1,^[^ ^41^ ^]^ and the eraser ALKBH5,^[^ ^42^ ^]^ is implicated in maintaining LCSC stemness. Previous reports have underscored the critical role of m^6^A modification in circRNAs biogenesis, function, and metabolism.^[^ ^6^ ^]^ However, how circRNAs are methylated and function in LCSCs are seldom reported. In this study, we observed increased expression of the m^6^A writer METTL3 in LCSCs, which promotes the m^6^A methylation of circRAPGEF1. m^6^A‐modified circRAPGEF1 was recognized and stabilized by m^6^A reader IGF2BP3. We also identified the m^6^A‐modified sites of circRAPGEF1 in HCC. Accumulating evidence suggests that non‐coding RNAs binding to IGF2BPs engages in cross‐talk with the regulation of mRNA stability. Our data revealed that circRAPGEF1 destabilizes *ASS1* mRNA through competitively binding to IGF2BP3, resulting in the downregulation of ASS1 expression in HCC cells.

Aspartate plays a crucial role in tumor cell survival, proliferation, and therapeutic resistance.^[^ ^11^ ^]^ ASS1, a key enzyme in aspartate metabolism, is associated with favorable prognosis in HCC.^[^ ^43^ ^]^ It has been demonstrated that ASS1 inhibits the proliferation and migration of HCC cells and enhances their sensitivity to systemic therapies.^[^ ^44^, ^45^ ^]^ However, the role of ASS1 in regulating HCC stemness has not been previously investigated. In the present study, we found that circRAPGEF1 reprograms aspartate metabolism in HCC by downregulating ASS1 expression, resulting in the accumulation of intracellular aspartate levels and the subsequent activation of the S6K/CAD signaling pathway. It has been established that CAD is the rate‐limiting enzyme that catalyzes aspartate participating in de novo pyrimidine synthesis,^[^ ^46^, ^47^ ^]^ which has been linked to enhanced tumor stemness properties.^[^ ^48^ ^]^ The increase in intracellular aspartate levels, which, under the catalysis of CAD, facilitating de novo pyrimidine synthesis, thus enhancing the stemness properties in HCC. This study elucidates the pivotal role of aspartate metabolic reprogramming in maintaining HCC stemness, paving the way for a promising therapeutic approach by targeting aspartate metabolism.

Sorafenib has been widely used as a first‐line systemic therapy for advanced HCC over the past two decades, but its clinical application is limited by low patient responsiveness.^[^ ^49^ ^]^ Thus, it is of paramount importance to identify new targets that can enhance the treatment sensitivity of sorafenib in HCC. In the present study, we found that high expression of circRAPGEF1 in HCC reduces the sensitivity of HCC cells to sorafenib, and knockdown of circRAPGEF1 sensitizes HCC cells to sorafenib. Targeting non‐coding RNAs in vivo, however, presents a significant challenge. The use of NPs for the delivery of siRNAs represents a feasible approach, as evidenced by our previous work in developing a range of NPs for siRNA delivery in the treatment of tumors.^[^ ^32^, ^33^, ^34^ ^]^ Herein, we employed Meo‐PEG‐S‐S‐PLA, a NP responsive to high glutathione levels in tumors, to deliver siRNAs targeting circRAPGEF1. The combined use of these NPs and sorafenib demonstrated a notable enhancement in therapeutic efficacy without significant toxicity in vivo. Our findings indicate that the use of circRAPGEF1‐targeting NP in conjunction with sorafenib represents a promising therapeutic strategy for HCC.

In summary, our study identified a novel circRNA, circRAPGEF1, in HCC and LCSCs. In LCSCs, circRAPGEF1 is upregulated through a METTL3/IGF2BP3‐mediated m^6^A modification mechanism. This upregulation leads to a reduction in *ASS1* mRNA stability, resulting in aspartate metabolic reprogramming and activation of the S6K/CAD signaling pathway. The activation of METTL3/circRAPGEF1/IGF2BP3/ASS1 axis results in enhanced stemness properties of HCC cells as well as poor prognosis. Targeting circRAPGEF1 with NPs enhances the sensitivity of HCC cells to sorafenib. Thus, our study offers a novel perspective on the treatment of HCC.

## 4 Experimental Section

### Clinical Samples

This study encompassed a total of 108 patients with HCC who underwent surgery at Sun Yat‐Sen Memorial Hospital, Sun Yat‐Sen University (Guangzhou, China). Cohort 1 comprised 30 pairs of frozen HCC and NATs, while cohort 2 consisted of 74 frozen HCC tissues complete with clinical information, which were used for RNA extraction and qRT‐PCR. Additionally, three surgically resected fresh HCC tissues were utilized for circRNA microarray assay, and one was employed for constructing the PDX model. The collection of clinical samples was in accordance with the Declaration of Helsinki, and approved by the ethics committees of Sun Yat‐Sen Memorial Hospital (SYSKY‐2024‐542‐01). Informed consent was obtained from all patients before sample collection.

### Experimental Animals

The 5–6‐week‐old BALB/c‐Nude and NOD/ShiLtJGpt (NCG) male mice utilized in this study were procured from GemPharmatech (Guangdong, China) and housed in a barrier environment at the Laboratory Animal Center of Sun Yat‐sen University. All experiments were approved by the Institutional Animal Care and Use Committee of Sun Yat‐sen University (SYSU‐IACUC‐2023‐000009) and followed the Guide for the Care and Use of Laboratory Animals.

### Isolation of CD133+ and CD133‐ HCC Cells

Fresh HCC tissues from three patients were stored on ice and transferred to the laboratory within 30 min after surgical excision. The tissues were minced and dissociated into single‐cell suspensions using the Tumor Dissociation Kit human (Miltenyi, Germany) and the gentleMACS Dissociator (Miltenyi). Live HCC cells were isolated by magnetic beads using the Tumor Cell Isolation Kit human (Miltenyi) and the Dead Cell Removal Kit (Miltenyi). Subsequently, CD133+ and CD133‐ HCC cells were sorted by magnetic beads using the CD133 MicroBead Kit‐Tumor Tissue human kit (Miltenyi).

### Aspartate Assay

Aspartate assay kit (Abcam, UK) was utilized to measure the intracellular aspartate concentration, following the manufacturer's instructions. One million HCC cells were subjected to ultrasonic digestion and homogenization in 200 µL of Aspartate Assay Buffer, and were centrifugated at 12 000 g for 10 min to collect supernatant. The supernatant was added to the Reaction Mix and incubated for 30 min. The absorbance was measured at 570 nm, and the corresponding aspartate concentration was calculated according to the standard curve.

### Statistical Analysis

Statistical analysis of this study was performed using GraphPad Prism 8.0 software. The proportion of CSCs in HCC was calculated through the Extreme Limiting Dilution Analysis (ELDA) software.^[^ ^50^ ^]^ Quantitative data were presented as mean and SD with at least three independent replicate experiments, and Student's *t*‐test, Mann–Whitney U test, one‐way ANOVA test, *χ* ^2^ test were used according to the type and normality of the data. Prognostic analysis of HCC patients was performed using Kaplan–Meier method and log‐rank test. *p* < 0.05 was considered statistically significant.

Additional methodology is provided in the Supporting Information.

## Conflict of Interest

The authors declare no conflict of interest.

## Author Contributions

J.S., S.M., Y.Y., and S.L. contributed equally to this work. X.X., Z.Z., and Z.X. designed the study. J.S., S.M., H.L., and H.L. participated in the in vitro and in vivo experiments, while S.L. performed NPs experiments. Y.Y., W.W., and C.H. collected and analyzed the clinical data. K.W., M.T., and J.W. performed the bioinformatics and statistical analysis. J.S. and Z.Z. were responsible for writing the manuscript, and all authors have reviewed and approved the submitted version.

## Supporting information



Supporting Information

## Data Availability

The data that support the findings of this study are available from the corresponding author upon reasonable request.
