# Supplementary material for: m6A‐Modified circRAPGEF1 Interaction with IGF2BP3 Promotes Hepatocellular Carcinoma Progression via Reprogramming Aspartate Metabolism
Source: Adv Sci (Weinh). 2025 Aug 13;12(41):e03851. doi: 10.1002/advs.202503851 (PMC12591180; doi:10.1002/advs.202503851)
Supplement: Supplementary file 1 — Supporting Information [file ADVS-12-e03851-s001.docx]

**Supporting Information**

**Supplementary Figures**


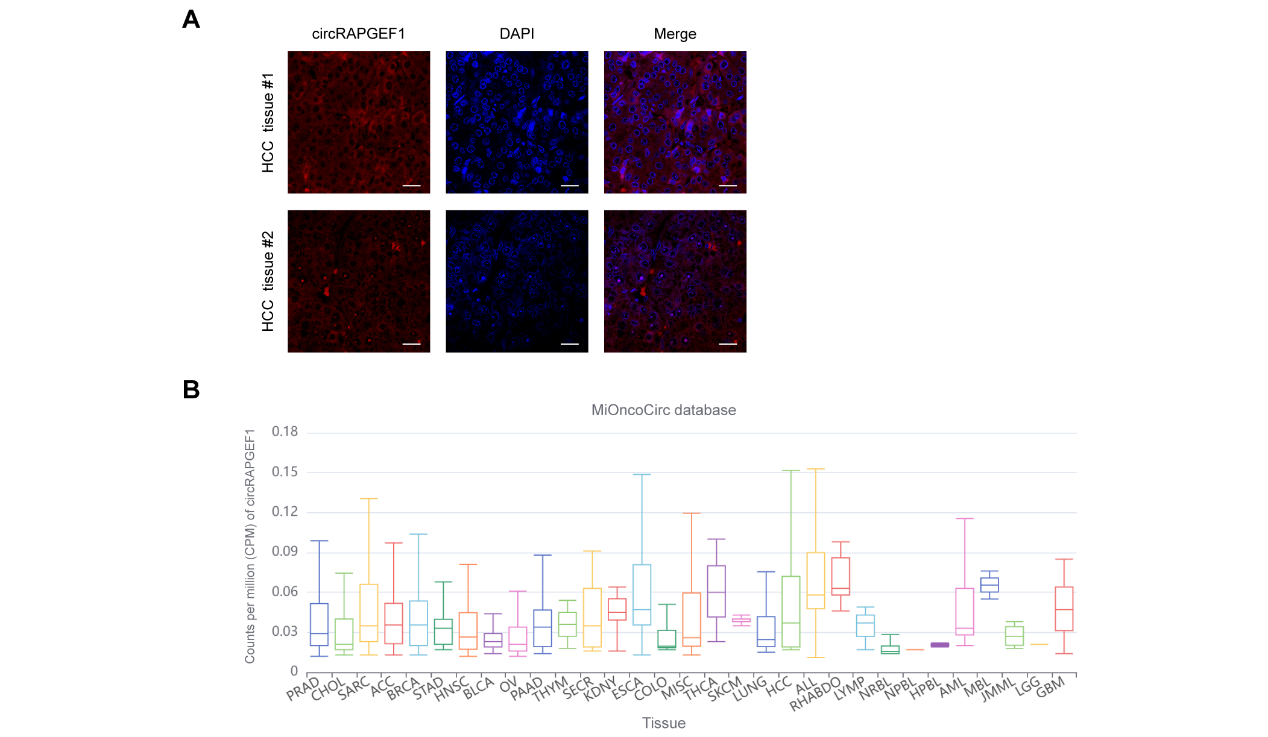


**Figure S1.** Expression level of circRAPGEF1 in HCC. A) Representative FISH images of circRAPGEF1 in HCC tissues. Scale bar: 10 μm. B) The expression pattern of circRAPGEF1 in pan-cancer types in the MiOncoCirc database.

**
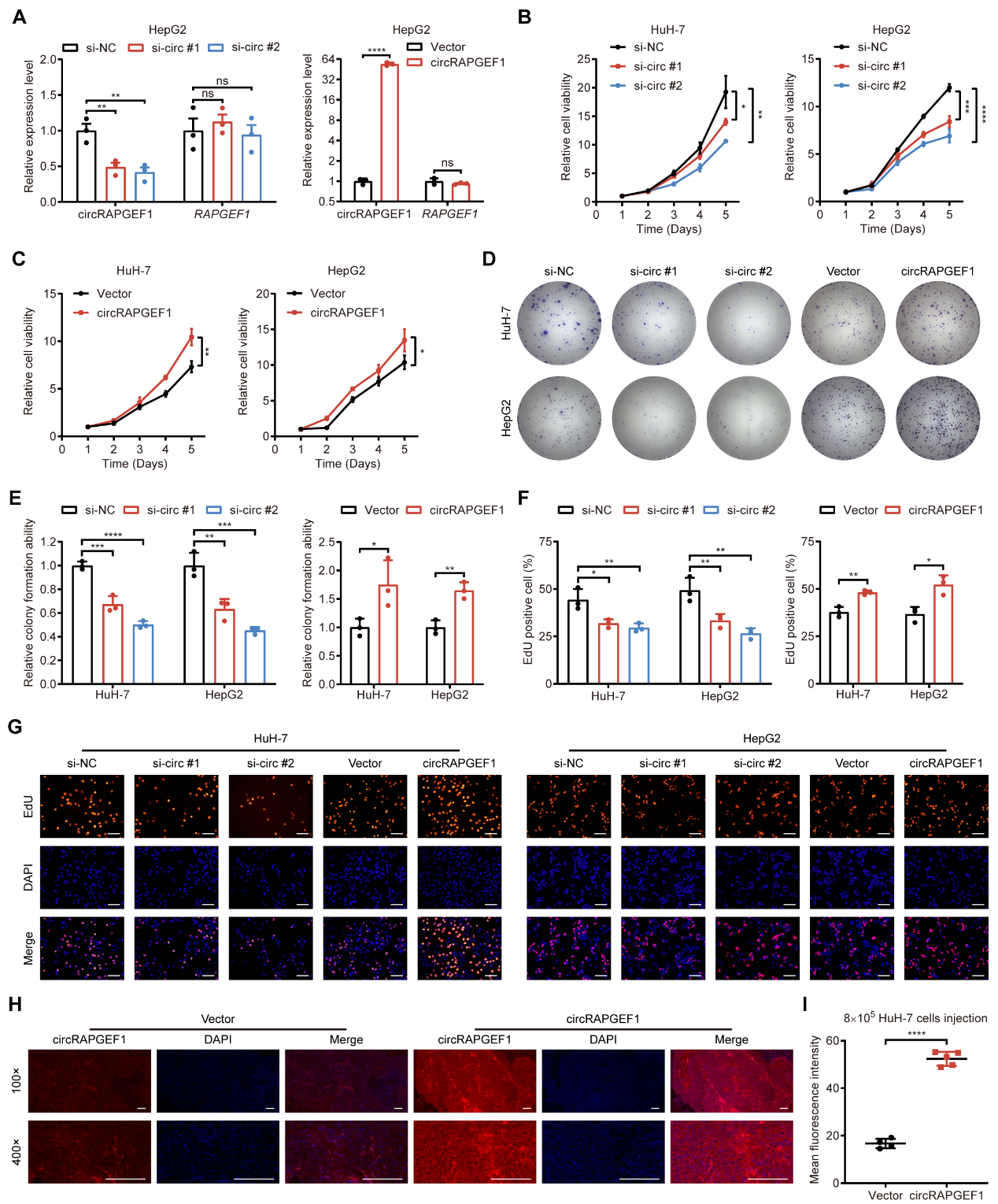
**

**Figure S2.** circRAPGEF1 promotes proliferation capacity of HCC cell. A) qRT-PCR analysis of circRAPGEF1 and RAPGEF1 expression in HepG2 cells transfected with control or circRAPGEF1-targeting siRNAs and Vector or circRAPGEF1 overexpression lentivirus. B, C) CCK-8 assay determined cell viability of HCC cells with circRAPGEF1silencing or overexpression. D, E) Representative images and quantification of colony formation assays in HCC with the indicated treatments. F and G) Quantification and representative images of EdU-labeled assays of HCC cells with the indicated treatments. Scale bar: 100μm. H and I) Representative images and quantification of circRAPGEF1 in FISH analysis in tumor specimens. Scale bar: 100μm. Data are presented as mean ± SD and analyzed by Student's t test or one‐way ANOVA with Tukey's multiple comparison test. ** P <* 0.05; *** P <* 0.01; **** P <* 0.001; ***** P <* 0.0001; ns: not signiﬁcant.


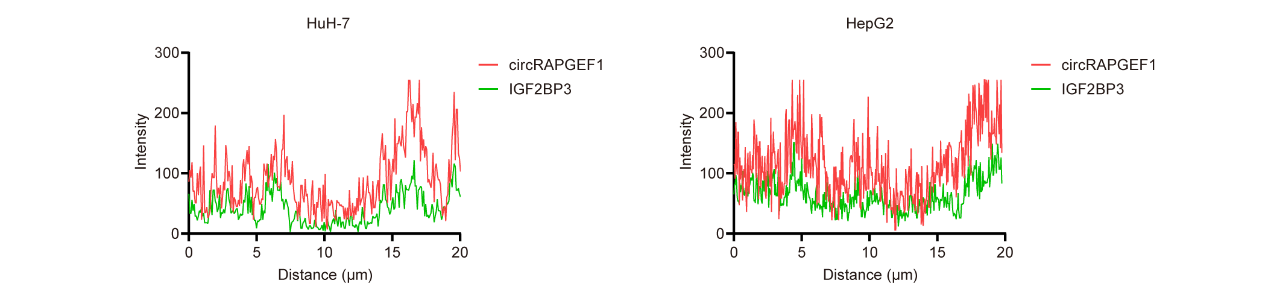


**Figure S3.** Quantitative analysis of FISH and IF shows circRAPGEF1 co-located in the cytoplasm with IGF2BP3 in HCC cells.

**
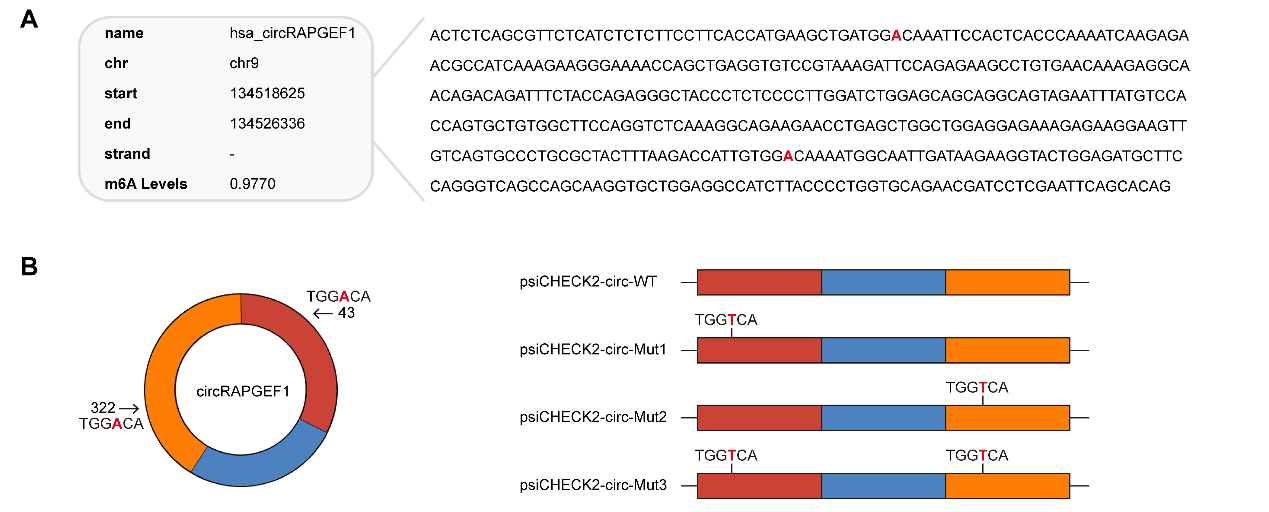
**

**Figure S4.** Analysis of the m^6^A modification in circRAPGRF1. A) circBank database showed m^6^A modification in circRAPGRF1. B) Schematic diagram of mutant m^6^A sites in circRAPGEF1 sequence.

**
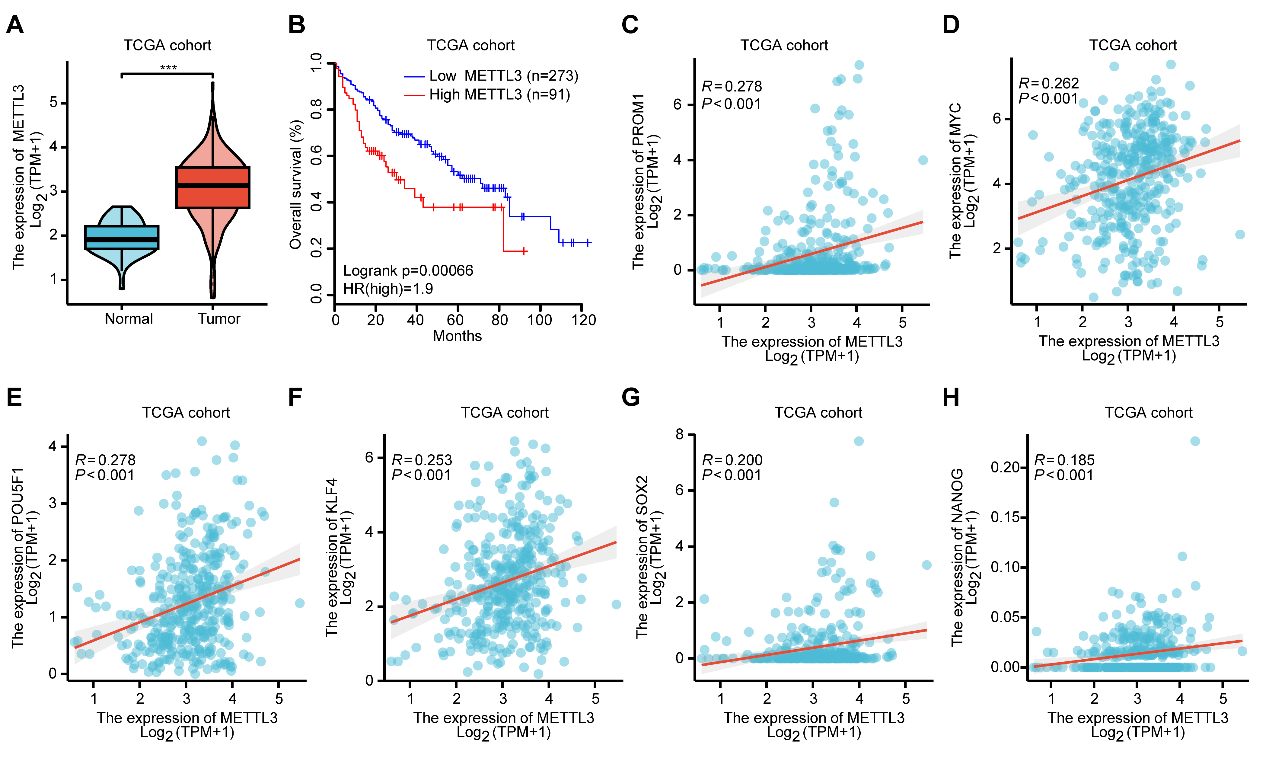
**

**Figure S5.** METTL3 Correlates with stemness markers in HCC. A, B) Boxplots illustrating METTL3 expression and K-M curve analysis of OS in the TCGA-LIHC cohort. C-H) Correlation analysis of METTL3 and PROM1, MYC, POU5F1, KLF4, SOX2 and NANOG expression in the TCGA-LIHC cohort. **** P <* 0.001.


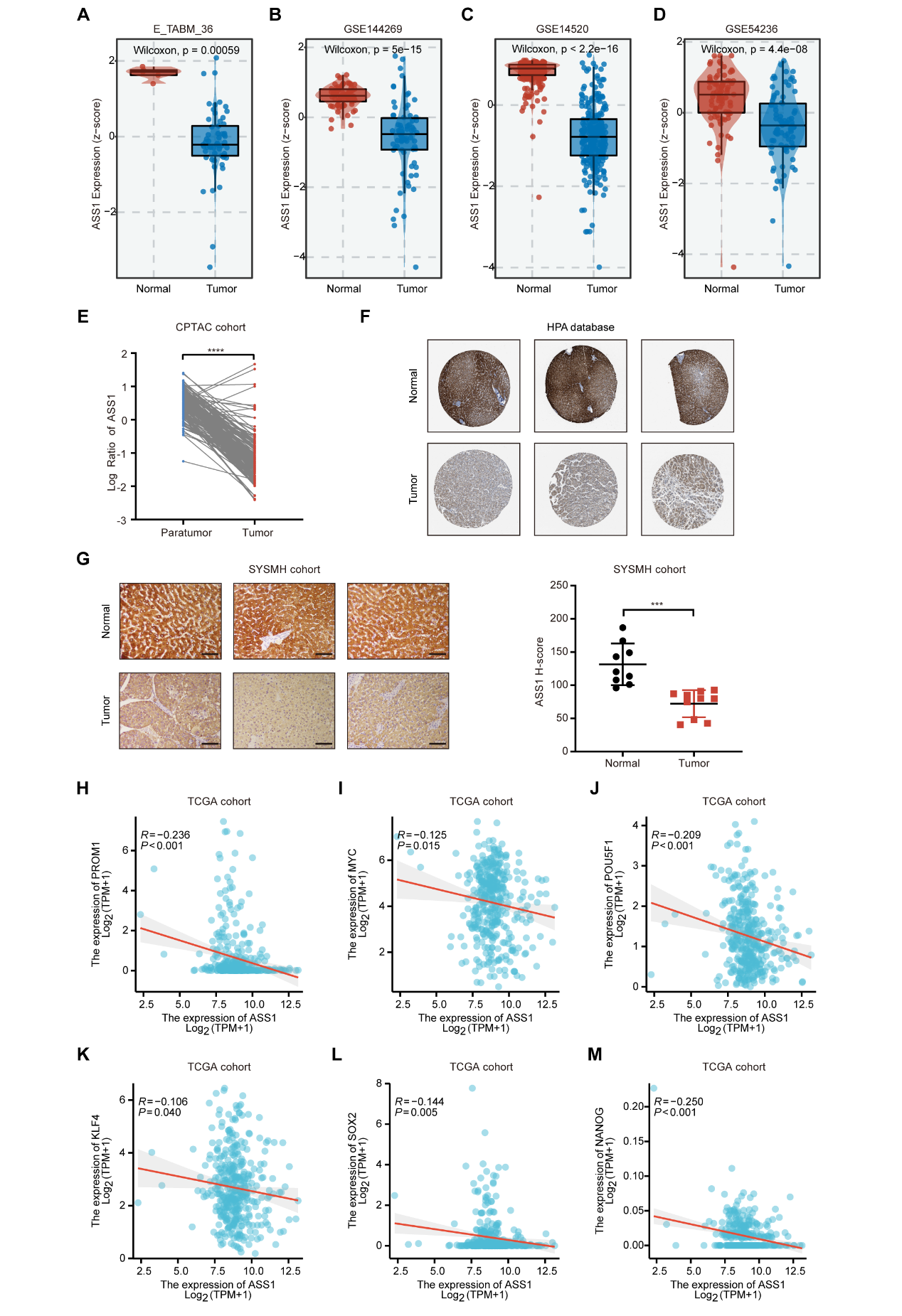


**Figure S6.** Multi-omics analysis of ASS1 expression in HCC. A-D) Boxplots illustrating the ASS1 expression in HCC and normal liver tissues across various cohort. E) Detection of ASS1 by mass spectrometry in the CPTAC cohort. F) Representative images of ASS1 staining of by IHC in the HPA cohort. G) Representative images and quantifications of ASS1 staining of by IHC in the SYSMH cohort. H-M) Correlation analysis of ASS1 with PROM1, MYC, POU5F1, KLF4, SOX2 and NANOG expression in TCGA-LIHC cohort. Data are analyzed by Student's t test. **** P <* 0.001; ***** P <* 0.0001.


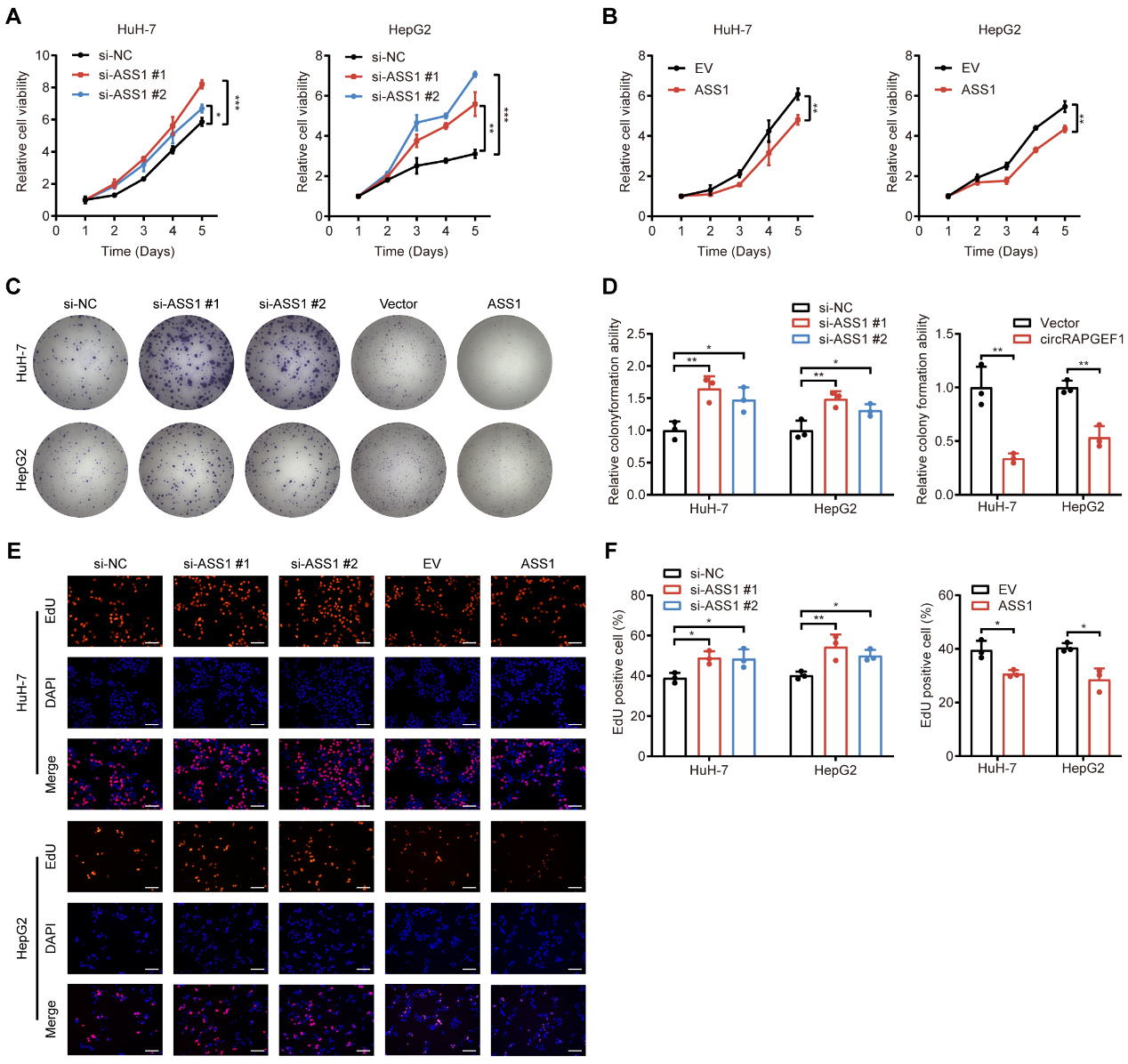


**Figure S7.** ASS1 suppressed cell proliferation in HCC. A, B) CCK-8 assay determined cell viability in HCC cells with ASS1 silencing or overexpression. C, D) Representative images and quantification of colony formation assays in HCC cells with the indicated treatments. E, F) Representative images and quantification of EdU-labeled assays in HCC cells with the indicated treatments. Scale bar: 100μm. Data are presented as mean ± SD and analyzed by Student's *t* test or one-way ANOVA with Tukey's multiple comparison test. ** P* < 0.05; *** P* < 0.01; **** P* < 0.001.


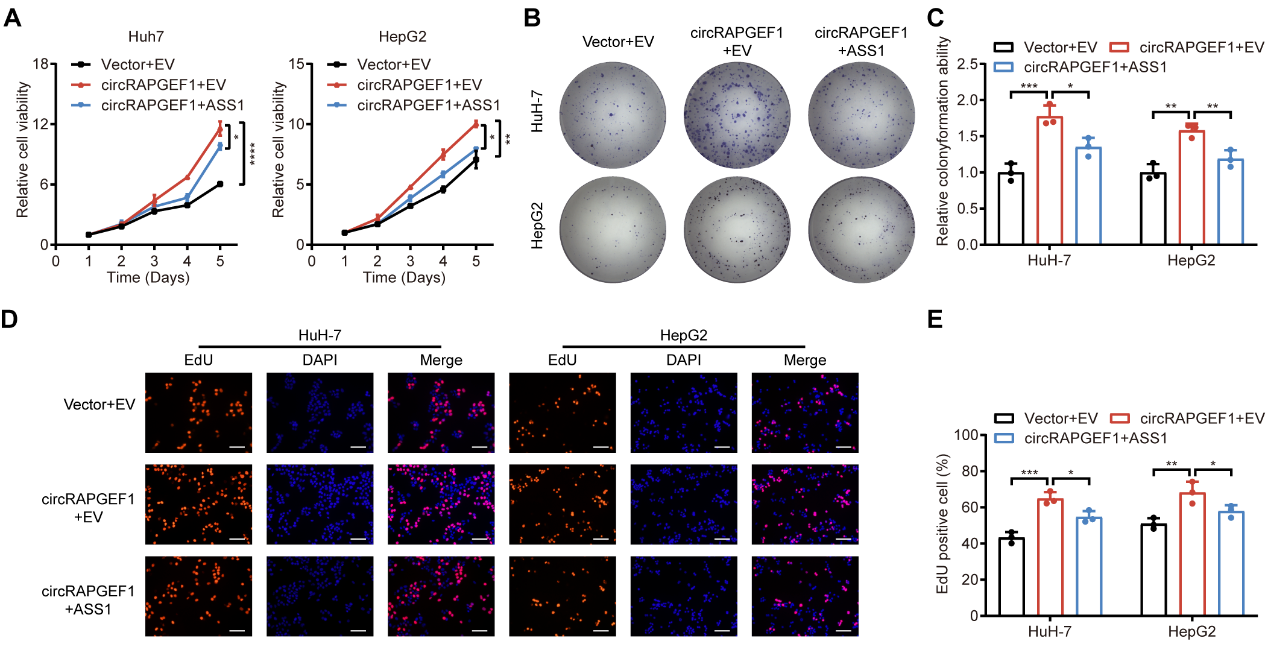


**Figure S8.** circRAPGEF1 downregulates ASS1 to enhance cell proliferation in HCC. A) CCK-8 assay determined cell viability in HCC cells co-transfected with vector/circRAPGEF1 overexpression lentivirus and EV/ASS1 overexpression plasmid. B, C) Representative images and quantification of colony formation assays in HCC cells with the indicated treatments. D, E) Representative images and quantification of EdU-labeled assays in HCC cells with the indicated treatments. Scale bar: 100μm. Data are presented as mean ± SD and analyzed by one-way ANOVA with Tukey's multiple comparison test. ** P* < 0.05; *** P* < 0.01; **** P* < 0.001; ***** P* < 0.0001; ns: not signiﬁcant.


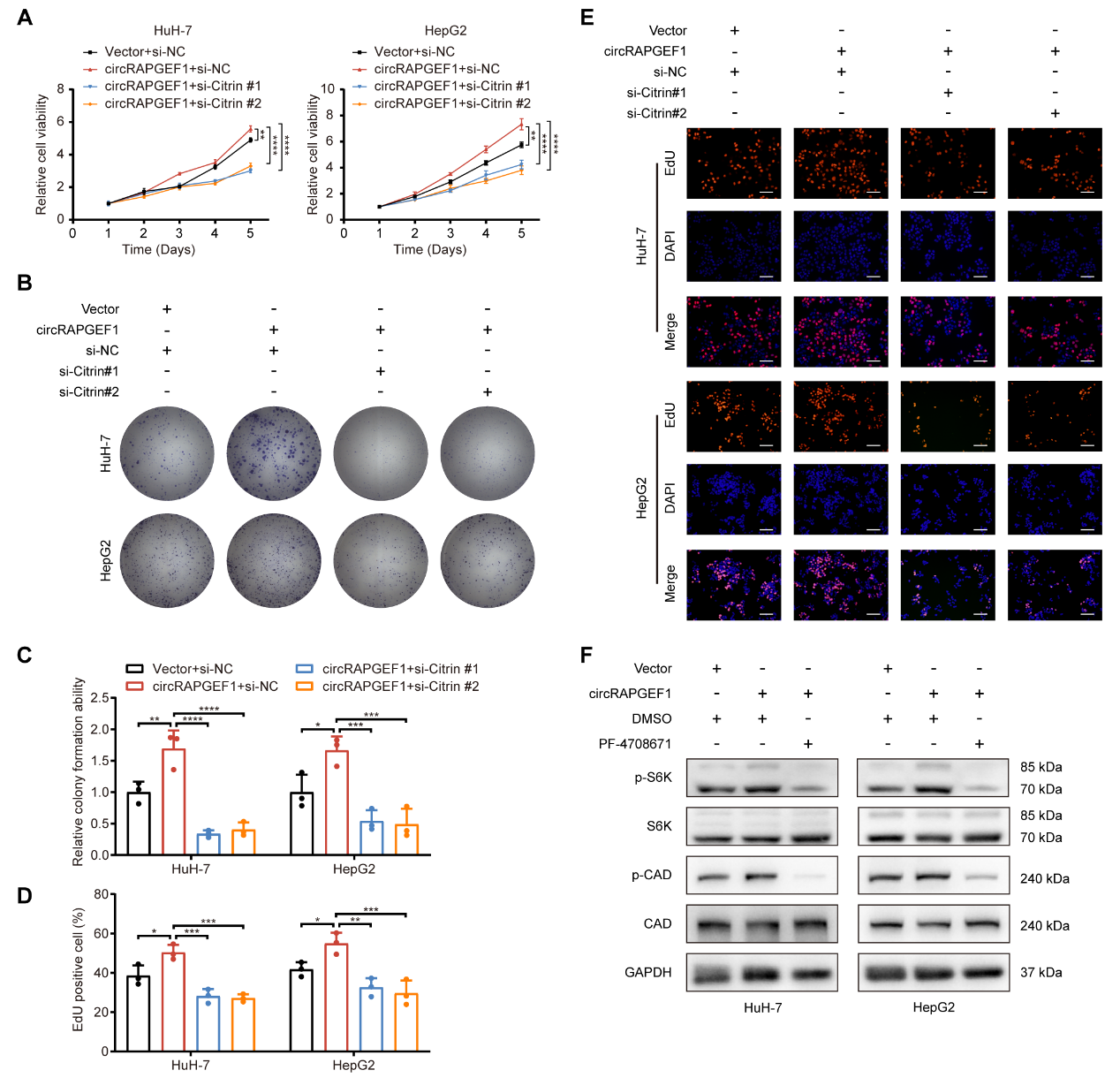


**Figure S9.** circRAPGEF1 upregulates aspartate levels to enhance cell proliferation in HCC cells. A) CCK-8 assay determined cell viability in HCC cells co-transfected with vector/circRAPGEF1 overexpression lentivirus and negative control/*Citrin*-targeting siRNAs. B, C) Representative images and quantification of colony formation assays in HCC cells with the indicated treatments. D, E) Quantification and representative images of EdU-labeled assays in HCC cells with the indicated treatments. Scale bar: 100μm. F) Western blot analysis of p-S6K, S6K, p-CAD, and CAD expression in HCC cells transfected with vector/circRAPGEF1 overexpression lentivirus and treated with DMSO or PF-4708671. Data are presented as mean ± SD and analyzed by one-way ANOVA with Tukey's multiple comparison test. ** P* < 0.05; *** P* < 0.01; **** P* < 0.001; ***** P* < 0.0001; ns: not signiﬁcant.


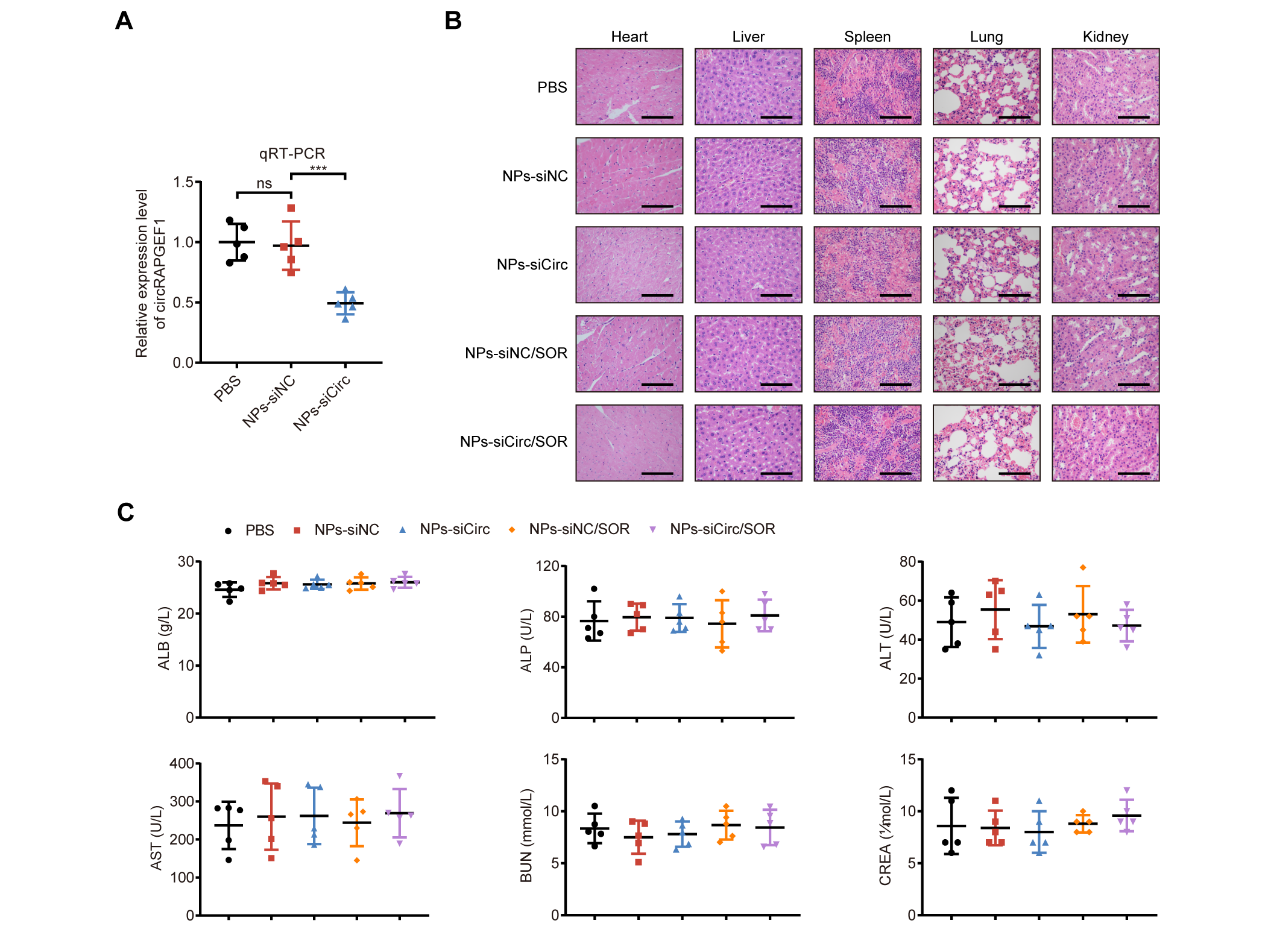


**Figure S10.** NPs treatments demonstrate favorable biocompatibility *in vivo*. A) qRT-PCR analysis of circRAPGEF1 expression in in tumor specimens of mice treated with PBS, NPs-siNC, and NPs-siCirc. B) Representative images of H&E staining in major organs of mice treated with PBS, NPs-siNC, NPs-siCirc, NPs-siNC/SOR and NPs-siCirc/SOR. C) Boxplots illustrating serum levels of ALB, ALP, AST, ALT, BUN and CREA of mice with the indicated treatments. Data are presented as mean ± SD and analyzed by one-way ANOVA with Tukey's multiple comparison test. **** P* < 0.001; ns: not signiﬁcant.

**Supplementary Tables**

**Table S1**. Associations between clinicopathological characteristics and circRAPGEF1 expression level in HCC patients.

| **Characteristics** |  | | **circRAPGEF1 expression** | | |
| --- | --- | --- | --- | --- | --- |
|  |  | **Low** | | **High** | ***p-*value** |
| **Total case** |  | 37 | | 37 |  |
| **Age (years)** |  |  | |  | 0.600 |
|  | ≤55 | 28 | | 26 |  |
|  | >55 | 9 | | 11 |  |
| **Gender** |  |  | |  | 0.691 |
|  | Female | 4 | | 3 |  |
|  | Male | 33 | | 34 |  |
| **HBV infection** |  |  | |  | 0.207 |
|  | Negative | 4 | | 8 |  |
|  | Positive | 33 | | 29 |  |
| **AFP (ng/mL)** |  |  | |  | 0.099 |
|  | ≤400 | 18 | | 25 |  |
|  | >400 | 19 | | 12 |  |
| **Tumor size (cm)** |  |  | |  | **0.032** |
|  | ≤5 | 19 | | 10 |  |
|  | >5 | 18 | | 27 |  |
| **Vescular invasion** |  |  | |  | 0.183 |
|  | Absent | 30 | | 25 |  |
|  | Present | 7 | | 12 |  |
| **Histologic grade** |  |  | |  | 0.586 |
|  | Ⅰ | 13 | | 9 |  |
|  | Ⅱ | 8 | | 10 |  |
|  | Ⅲ | 16 | | 18 |  |
| **TNM Stage** |  |  | |  | **0.012** |
|  | I-II | 13 | | 4 |  |
|  | III-IV | 24 | | 33 |  |

**Table S2**. The top 10 highest scores for specific proteins pulled down by circRAPGEF1 probe in mass spectrometry analysis.

| **Protein** | **Score** | **Coverage (%)** | **Molecular weight (kDa)** |
| --- | --- | --- | --- |
| G3BP1 | 457 | 26.2 | 52.2 |
| SLC25A13 | 394 | 21.5 | 74.5 |
| G3BP2 | 347 | 26.3 | 54.1 |
| ABCD3 | 276 | 15.9 | 75.9 |
| HSD17B4 | 272 | 12.9 | 80.1 |
| ATAD3A | 252 | 17.7 | 71.6 |
| TRIP4 | 230 | 13.8 | 67.0 |
| SYNCRIP | 218 | 14.6 | 69.8 |
| FUS | 204 | 12 | 53.6 |
| **IGF2BP3** | **183** | **19.3** | **64.0** |

**Table S3**. The list for antibodies and dilution concentrations used in this study.

| **Antibody** | **Supplier** | **Catalog** | **Application** | **Dilution** |
| --- | --- | --- | --- | --- |
| GAPDH | Ray antibody | RM2002 | WB | 1:5000 |
| CD133 | CST | 64326 | WB  IHC | 1:1000  1:200 |
| Ki67 | Servicebio | GB111499 | IHC | 1:800 |
| ASS1 | Proteintech | 16210-1-AP | WB  IHC | 1:1000  1:400 |
| IGF2BP3 | Proteintech | 14642-1-AP | WB  IF  RIP | 1:1000  1:200  5 μg |
| N6-methyladenosine | Abcam | ab208577 | RIP | 5 μg |
| IgG | Proteintech | B900610 | RIP | 5 μg |
| Phospho-p70 S6K(Thr389) | Proteintech | 28735-1-AP | WB | 1:1000 |
| p70 S6K | Proteintech | 14485-1-AP | WB | 1:1000 |
| Phospho-CAD (Ser1859) | Affinity Biosciences | AF4415 | WB | 1:1000 |
| CAD | Affinity Biosciences | DF7658 | WB | 1:1000 |
| Rabbit IgG, HRP-linked | CST | 7074 | WB | 1:3000 |
| Mouse IgG, HRP-linked | CST | 7076 | WB | 1:3000 |
| Dylight 488, Goat Anti-Rabbit IgG | Abbkine | A23220 | IF | 1:200 |
| HRP-labeled Goat Anti-Rabbit IgG(H+L) | Beyotime | A0208 | IHC | 1:50 |

**Table S4**. The list for primers used in this study.

| **Primers** | **Sequence (5’-3’)** |
| --- | --- |
| circRAPGEF1 Forward | TGGAGGCCATCTTACCCCTG |
| circRAPGEF1 Reverse | TTTACGGACACCTCAGCTGG |
| circ89252 Forward | GTCATCAAGGCTGTGCTGGA |
| circ89252 Reverse | TGGCGTTCTCTTGATTTTGGG |
| *RAPGEF1* Forward | TGCCGACACATTCAAGAAGCGC |
| *RAPGEF1* Reverse | GGAAGACCAGTTCCATCAGCAG |
| *GAPDH* Forward | CAAGGCTGAGAACGGGAAG |
| *GAPDH* Reverse | TGAAGACGCCAGTGGACTC |
| *IGF2BP3* Forward | TCGTGACCAGACACCTGATGAG |
| *IGF2BP3* Reverse | GGTGCTGCTTTACCTGAGTCAG |
| *METLL3* Forward | GGACACCAACAACTTCTTCGGC |
| *METLL3* Reverse | GTCGGTCATATTTTTCAGCACGTC |
| *METTL14* Forward | CTGAAAGTGCCGACAGCATTGG |
| *METTL14* Reverse | CTCTCCTTCATCCAGATACTTACG |
| *WTAP* Forward | GCAACAACAGCAGGAGTCTGCA |
| *WTAP* Reverse | CTGCTGGACTTGCTTGAGGTAC |
| *KIAA1429* Forward | TGACCTTGCCTCACCAACTGCA |
| *KIAA1429* Reverse | AGCAACCTGGTGGTTTGGCTAG |
| *OCC1* Forward | CCAAGGAAATTCTAAACAGTCACC |
| *OCC1* Reverse | CCACAGAAAGTTCCATAAACAAGTG |
| *ASS1* Forward | GCTGAAGGAACAAGGCTATGACG |
| *ASS1* Reverse | GCCAGATGAACTCCTCCACAAAC |
| *Citrin* Forward | AGATGGTTCGGTCCCACTTGCA |
| *Citrin* Reverse | ACCAGTGGTGATTTCTCCTGCC |
| *CAD* Forward | TAGTCCTTGGCTCTGGCGTCTA |
| *CAD* Reverse | TAGTCGGTGCTGACTGTCTCTG |
| *U6* Forward | CGCTTCGGCAGCACATATAC |
| *U6* Reverse | TTCACGAATTTGCGTGTCAT |
| *18S* Forward | GGAGTATGGTTGCAAAGCTGA |
| *18S* Reverse | TCCTGCTTTGGGGTTCGATT |

**Table S5**. The list for RNA oligonucleotides used in this study.

| **Oligonucleotides** | **Sequence (5’-3’)** |
| --- | --- |
| **siRNAs** |  |
| si-NC sense | UUCUCCGAACGUGUCACGUTT |
| si-NC antisense | ACGUGACACGUUCGGAGAATT |
| si-circRAPGEF1#1 sense | CUCGAAUUCAGCACAGACUCUTT |
| si-circRAPGEF1#1 antisense | AGAGUCUGUGCUGAAUUCGAGTT |
| si-circRAPGEF1#2 sense | CGAAUUCAGCACAGACUCUTT |
| si-circRAPGEF1#2 antisense | AGAGUCUGUGCUGAAUUCGTT |
| si-IGF2BP3#1 sense | GGAUUCGGAAACUUCAGAUTT |
| si-IGF2BP3#1 antisense | AUCUGAAGUUUCCGAAUCCTT |
| si-IGF2BP3#2 sense | GCUGCUGAGAAGUCGAUUATT |
| si-IGF2BP3#2 antisense | UAAUCGACUUCUCAGCAGCTT |
| si-METTL3#1 sense | GCUACCUGGACGUCAGUAUTT |
| si-METTL3#1 antisense | AUACUGACGUCCAGGUAGCTT |
| si-METTL3#2 sense | GGUGACUGCUCUUUCCUUATT |
| si-METTL3#2 antisense | UAAGGAAAGAGCAGUCACCTT |
| si-ASS1#1 sense | GAACAAGGCUAUGACGUCATT |
| si-ASS1#1 antisense | UGACGUCAUAGCCUUGUUCTT |
| si-ASS1#2 sense | CUGACAUUCUCGAGAUCGATT |
| si-ASS1#2 antisense | UCGAUCUCGAGAAUGUCAGTT |
| si-Citrin#1 sense | GCCCUUUAACUUGGCUGAGGTT |
| si-Citrin#1 antisense | CCCAGACCAAACCUGUAGGCTT |
| si-Citrin#2 sense | GUAUAGAGGUCUGUUGCCATT |
| si-Citrin#2 antisense | UGGCAACAGACCUCUAUACTT |
| **sh-RNAs** |  |
| sh-circ #2 | CCGGCGAATTCAGCACAGACTCTCTCGAGAGAGTCTGTGCTGAATTCGTTTTTGAATT |
| **NP-siCirc** |  |
| sense | CGAAUUCAGCACAGACUCUTT |
| antisense | AGAGUCUGUGCUGAAUUCGTT |
| **Probes for FISH** |  |
| Cy3-*U6* | TTTGCGTGTCATCCTTGCG |
| Cy3-*18S* | CTTCCTTGGATGTGGTAGCCGTTTC |
| Cy3-circRAPGEF1 | GAUGAGAACGCUGAGAGUCUGUGCUGAAUUCGAGGA |
| **Probes for RNA pull-down** |  |
| Biotin-NC | UCCUCGAAUUCAGCACAGACUCUCAGCGUUCUCAUC |
| Biotin-circRAPGEF1 | GAUGAGAACGCUGAGAGUCUGUGCUGAAUUCGAGGA |

**Supplementary methods**

*Cell lines*: The human HCC cell line HuH-7 was procured from the Cell Bank of the Type Culture Collection of the Chinese Academy of Sciences. The human HCC cell line HepG2 and embryonic kidney cell line HEK293T were procured from Zhong Qiao Xin Zhou Biotechnology (Shanghai, China). HuH-7, HepG2, and HEK-293T cells were cultured in DMEM high glucose medium (Gibco, USA) supplemented with 10% fetal bovine serum (Gibco), 100 U/mL penicillin and 100 μg/mL streptomycin. All cells were cultured in an incubator maintained at 37℃ and 5% CO_2_. The authenticity of all cell lines was confirmed through short tandem repeat profiling, and the absence of mycoplasma was verified.

*Animal models*: For the *in vivo* limiting dilution assay，2×10^5^, 4×10^5^ and 8×10^5^ of Huh-7 cells were subcutaneously injected into the right flank of BALB/c Nude mice (n = 5 per group). Tumor volume was recorded every week. Mice were sacrificed 8 weeks after injection.

For the PDX models, fresh HCC tissue sample was obtained from an HCC patient who underwent radical hepatectomy. The tumor tissue was cut into small pieces and implanted subcutaneously into NCG mice (F1). When the tumors volume exceeded 1500 mm³, the tumors were excised into equal volumes and implanted into the next generation of mice. When volumes of F3 PDXs reached approximately 60 mm³, the mice were randomly divided into two groups (n = 5 per group) and injected intratumorally with negative control or sh-circRAPGEF1 lentivirus. Tumor volume was recorded every three days, and the tumor volume was calculated as length × width × width / 2. Mice were sacrificed 25 days after implantation.

For the *in vivo* nanoparticles (NPs) treatment, 3×10^6^ of Huh-7 cells were subcutaneously injected into the right flank of BALB/c Nude mice. Once volumes of tumors reached approximately 60 mm³, the mice were randomly divided into five groups (n=5 per group). The combination therapy utilized siRNA-loaded NPs and sorafenib. The mice were intravenously injected with PBS or NPs (1 nmol of siRNAs per mouse) every two days for three times and were intragastric administered with 0.5% CMC-Na or sorafenib (20 mg/kg) once daily. The tumor volume was recorded every three days, and mice were sacrificed 18 days after the start of treatment at the humane endpoint.

At the end of each *in vivo* experiments, the subcutaneous tumor tissues were meticulously excised, photographed, and weighed. Subsequently, half of these tissues were paraformaldehyde-fixed, paraffin-embedded, and followed by histopathological examination. The remaining specimens were stored in a -80℃ freezer. The blood was collected and the serum was isolated for further analysis of multiple blood indicators.

*circRNA microarray*: Three pairs of primary CD133+ and CD133- HCC cells were lysed with TRIzol reagent (Invitrogen, USA) to extract total RNA. The RNA was then digested with RNase R to remove linear RNA and enrich for circRNAs. The enriched circRNAs were amplified, transcribed, and then detected with the Arraystar Human circRNA Array V2 (8x15K, Arraystar, USA), followed by the bioinformatics analysis, performed by Aksomics (Shanghai, China). Differentially expressed circRNAs were defined as *p*-value < 0.05 and |fold change (FC)| >1.5.

*Sphere-formation assay*: A total of 1×10^3^ HuH-7 or HepG2 cells were inoculated in 96-well ultra-low attachment cell culture plates (Corning, USA) and cultured using DMEM/F12 medium (Gibco) with 20 ng/ml bFGF (PeproTech, USA), 20 ng/ml EGF (PeproTech), and B27 Supplement (Gibco). After 10 days, the number of spheres (diameter > 50 mm) was quantified under a microscope.

*mRNA sequencing*: The Vector and circRAPGEF1-overexpression HepG2 cells, with three independent replicates each, were lysed with Trizol reagent (Invitrogen) to extract total RNA. In brief, mRNA was enriched with capture beads, synthesized into cDNA, amplified, purified, and cDNA libraries were constructed. The mRNA sequencing was performed using the Novaseq Sequencing Platforms (Illumina, USA), and the assays and analyses were administered by IGE Biotechnology (Guangzhou, China). Differentially expressed genes (DEGs) were defined as *p*-value < 0.05 and |FC| >2.

*Protein extraction and Western Blot*: RIPA buffer supplemented with protease and phosphatase inhibitor cocktail (Bimake, USA) was used to lyse cells, and bicinchoninic acid method were employed to prepare total protein samples. The samples were separated through a 10% SDS-PAGE gel in Tris-Glycine buffer, and were transferred to a PVDF membrane. After incubation with primary antibodies and HRP-conjugated secondary antibodies, protein bands were visualized using Immobilon Western HRP Substrate (Merck Millipore) and imaged with a SmartChemi Integrated Imaging and Analysis System (SinSage, China). GAPDH was used as a loading control. The details of antibodies used are provided in Supplementary Table 3.

*RNA extraction, reverse transcription, and qRT-PCR assay*: In brief, HCC cells and tissues were lysed by TRIzol reagent (Invitrogen) for RNA extraction and subsequent cDNA synthesis using HiScript III RT SuperMix (Vazyme, China) according to the manufacturer's instructions. qRT-PCR was applied to detect the level of RNA expression using the ChamQ Universal SYBR qPCR Master Mix (Vazyme), which was detected by a QuantStudio Real-Time PCR System (Applied Biosystems, USA). *GAPDH* was used as an endogenous reference to quantify relative expression of target genes using the 2^-∆∆Ct method. The primers utilized in this study are listed in Supplementary Table 4.

Specifically, for the reverse transcription assay, oligo dT primer and random 6 mer primer (AG Bio, China) were used to discriminate the structurally distinct between mRNAs and circRNAs. In the RNA degradation assay, total RNA was subjected to treatment with RNase R (Epicentre, USA) prior to reverse transcription in order to demonstrate the stability of circRNAs *in vitro*, as was previously reported^[1]^.

*RNA stability assay*: A total of 2×10^5^ HuH-7 and HepG2 cells were cultured in 6-well plates and treated with 5 μg/mL actinomycin D (AbMole, USA). The cells were harvested, and RNA was extracted at the multiple time points. Subsequently, qRT-PCR was employed to detect RNA expression at indicated time points. The RNA expression levels at each time point were then normalized to the expression levels at 0 hours.

*RNA subcellular isolation*: First, cytoplasmic and nuclear RNA were extracted from HuH-7 and HepG2 cells using the RNA Subcellular Isolation Kit (Active Motif, USA) according to the manufacturer's instructions. Subsequent reverse transcription and qRT-PCR) were used to determine RNA expression levels. *18S* and *U6* were used as endogenous references in the cytoplasm and nucleus, respectively. The relative RNA abundance in the cytoplasm and nucleus was calculated using the 2^-∆Ct method.。

*Fluorescence in situ hybridization (FISH) and immunofluorescence (IF)*: The RNA FISH kits and fluorescent probes were procured from Suzhou GenePharma (Jiangsu, China). The HuH-7 and HepG2 cells were cultured on confocal dishes. FISH were performed according to the manufacturer's instructions, specifically, the Cy3-labeled circRAPGEF1 probe was administered at a working concentration of 1 µM. For IF, the cells were blocked after FISH and then sequentially incubated with an IGF2BP3 antibody (Proteintech, China) and a FITC-labeled secondary antibody (Abbkine, China). The cells were then stained with DAPI. Finally, images were captured and merged by a confocal laser scanning microscope (Carl Zeiss, Germany). The list of antibodies and dilution are provided in Supplementary Table 3.

*siRNAs, plasmids and lentivirus infection*: In the present study, siRNAs for circRAPGEF1, IGF2BP3, METTL3, ASS1, Citrin, and negative control siRNAs were synthesized by Suzhou GenePharma (Guangdong, China), HuH-7 and HepG2 cells were transfected with siRNAs using Lipofectamine RNAiMAX reagent (Invitrogen). The RNA oligonucleotides utilized in this study are listed in Supplementary Table 5. Flag-tagged full-length, KH domain-depleted, and RRM domain-depleted IGF2BP3 were cloned into pCDH plasmid by Guangzhou IGE Biotechnology. The ASS1 expression plasmid was purchased from YouBio (Hunan, China) and cloned into the pcDNA3.1 and the pCDH plasmids. HuH-7, HepG2 and HEK293T cells were transfected with the aforementioned plasmids using the X-tremeGENE HP DNA transfection reagent (Roche, Germany). The circRAPGEF1 overexpressing (cloned into plenti-ciR plasmid) and silencing lentivirus (cloned into pLKO.1 plasmid) were supplied by Guangzhou IGE Biotechnology. The Vector and short hairpin negative control (sh-NC) lentivirus were used as control, respectively. To establish stable circRAPGEF1 overexpression and knockdown cells, the HuH-7 cells and HepG2 cells were infected with the lentivirus and polybrene, followed by selection with puromycin.

*Cell counting kit-8 (CCK-8) assays*: For cell proliferation assay, HuH-7 and HepG2 cells (1×10³ cells/well) were seeded in 96-well cell culture plates. For five consecutive days, cells were incubated with 10% CCK-8 (APExBIO, USA) in complete medium for 2 hours daily. Absorbance at 450 nm was measured using a microplate reader (Tecan, USA), and cell viability was normalized to the absorbance value of Day 1. For cell cytotoxicity assay, HuH-7 and HepG2 cells (5×10³ cells/well) were treated with sorafenib (Selleck, USA) at concentrations of 0, 5, 10, 15, 20, or 25 μM in 96-well plates (triplicate wells per concentration). After 48 hours of treatment, cell viability was assessed using the CCK-8 assay, with absorbance values normalized to those of the 0 μM sorafenib-treated control group.

*Proliferation assays*: For Colony Formation Assay, HuH-7 and HepG2 cells were seeded in 6-well plates at a density of 2×10³ cells/well and cultured for 14 days. Colonies were fixed with 4% paraformaldehyde (PFA), stained with 0.5% crystal violet, and washed with pure water. Visible colonies (>50 cells/colony) were imaged and quantified. For EdU incorporation assay, cells were incubated with 50 μM EdU (APExBIO) in complete medium for 2 hours at 37°C, followed by fixation with 4% PFA and permeabilization with 0.3% Triton X-100. The EdU Imaging Kits Cy3 (APExBIO) was used to detect EdU-positive nuclei according to the manufacturer's protocol. Nuclei were counterstained with DAPI (Solarbio, China), and images were captured using an inverted fluorescence microscope (Olympus, Japan). The EdU-positive rate was calculated as the percentage of Cy3-labeled cells relative to total DAPI-positive nuclei.

*RNA Pull-down*: Biotin-labeled circRAPGEF1 and negative control probes (sequences provided in Supplementary Table 5) were synthesized by Suzhou GenePharma (Jiangsu, China). Full-length and truncated IGF2BP3 expression plasmids were purchased from IGE Biotechnology. The RNA-protein interactions were analyzed using the Pierce Magnetic RNA-Protein Pull-Down Kit (Thermo Fisher Scientific Scientific, USA) according to the manufacturer’s protocol. Briefly, 1×10⁷ cells were harvested, lysed, and centrifuged to collect supernatants. Lysates were incubated with streptavidin magnetic beads pre-coated with 50 pmol probes overnight with rotation. The beads were then washed and bound proteins were eluted for subsequent experiments.

*Silver stain and mass spectrometry (MS) analysis*: First, protein samples enriched by RNA pull-down assays were resolved on SDS-PAGE gel. Subsequently, the gel underwent fixation, sensitization, washing, and staining using the Fast Silver Stain Kit (Beyotime, China) according to manufacturer’s instruction. Gel regions showing differential bands between circRAPGEF1 probes and negative controls were then excised, proceed to peptides, followed by MS analysis on a Q Exactive hybrid quadrupole-Orbitrap mass spectrometer (Thermo Fisher Scientific), performed by Guangzhou Fitgene Bio (Guangdong, China). The raw data were processed with MASCOT software by searching Uniprot database. Candidate circRAPGEF1-interacting proteins were provided in the Supplementary Table 2.

*RNA immunoprecipitation (RIP)*: In the present study, RIP assays were employed using the Magna RIP Kit (Merck Millipore, USA) according to the manufacturer’s guidelines. The details of antibodies used are provided in Supplementary Table 3. Briefly, 2×10⁷ HuH-7 and HepG2 cells were harvested, lysed in RIP lysis buffer containing protease inhibitor cocktail and RNase inhibitor, and centrifuged to collect supernatants. Supernatants were incubated with protein A/G magnetic beads pre-conjugated with antibodies (5 μg antibody per RIP) overnight with rotation. Beads were washed with wash buffer, followed by RNA extraction using TRIzol reagent(Invitrogen) and qRT-PCR analysis. RNA enrichment was normalized to the input and calculated as fold change relative to the IgG immunoprecipitates.

*Dual luciferase reporter gene assay*: In the present study, psi-CHECK-2 plasmids with wild-type and mutant circRAPGEF1 sequences were purchased from Suzhou Synbio Tech (Jiangsu, China). HEK293T cells were cultured in 12-well plate (1×10^5^ cells/well) and were transfected with either an IGF2BP3 overexpressing plasmid or an empty vector plasmid. Subsequently to the initial transfection, the cells were subjected to transfection with the indicated psi-CHECK-2 plasmids. Two days after the transfection，the cells were harvested and processed using the Dual Luciferase Reporter Gene Assay Kit (Yeasen Bio, China). The activity of firefly and Renilla luciferase was detected by a multimode microplate reader (Tecan).

*Preparation of siRNA-loaded NPs*: The siRNA-loaded NPs were synthesized following our previously developed protocol^[2]^. Briefly, Meo-PEG-S-S-PLGA polymer and G0-C14 were separately dissolved in N, N-dimethylformamide, combined with siRNA solution, and introduced into deionized water under vigorous stirring (1000 rpm). The mixture was then purified via an ultra-centrifugal filter, 100 kDa MWCO (Merk Millipore), washed with ultrapure water, and resuspended in PBS. The particle sizes of NPs were determined by dynamic light scattering (Malvern Panalytical, USA).

*Immunohistochemistry (IHC)*: Formalin-fixed, paraffin-embedded (FFPE) tissue sections were deparaffinized, rehydrated, subjected to antigen retrieval in preheated Tris-EDTA buffer (pH 9.0) and blockage of endogenous peroxidase activity. After incubation with primary antibodies and HRP-conjugated secondary antibodies, signal was stained using the DAB (Boster, China) under microscopic monitoring. was used to highlight cell nuclei. Nuclei were counterstained with hematoxylin (Servicebio, China), and images were captured using a microscope (Nikon, Japan). H-scores were analyzed using ImageJ software with IHC Profiler plugin^[3]^.

**References**

[1] Liu, H., Y. Yan, J. Lin, et al. “Circular RNA circSFMBT2 downregulation by HBx promotes hepatocellular carcinoma metastasis via the miR-665/TIMP3 axis.” *Molecular Therapy - Nucleic Acids*, no. 29 (2022): 788-802. https://doi.org/10.1016/j.omtn.2022.08.008.

[2] Li, S., L. Xu, G. Wu, et al. “Remodeling Serine Synthesis and Metabolism via Nanoparticles (NPs)‐Mediated CFL1 Silencing to Enhance the Sensitivity of Hepatocellular Carcinoma to Sorafenib.” *Advanced Science*, no. 10 (2023): e2207118. https://doi.org/10.1002/advs.202207118.

[3] Varghese, F., A. B. Bukhari, R. Malhotra, A. De. “IHC Profiler: an Open Source Plugin for the Quantitative Evaluation and Automated Scoring of Immunohistochemistry Images of Human Tissue Samples.” *PLoS One*, no. 9 (2014): e96801. https://doi.org/10.1371/journal.pone.0096801.
